# Antibiotic Modulation of Capsular Exopolysaccharide and Virulence in *Acinetobacter baumannii*

**DOI:** 10.1371/journal.ppat.1004691

**Published:** 2015-02-13

**Authors:** Edward Geisinger, Ralph R. Isberg

**Affiliations:** 1 Department of Molecular Biology and Microbiology, Tufts University School of Medicine, Boston, Massachusetts, United States of America; 2 Howard Hughes Medical Institute, Boston, Massachusetts, United States of America; Emory University School of Medicine, UNITED STATES

## Abstract

*Acinetobacter baumannii* is an opportunistic pathogen of increasing importance due to its propensity for intractable multidrug-resistant infections in hospitals. All clinical isolates examined contain a conserved gene cluster, the K locus, which determines the production of complex polysaccharides, including an exopolysaccharide capsule known to protect against killing by host serum and to increase virulence in animal models of infection. Whether the polysaccharides determined by the K locus contribute to intrinsic defenses against antibiotics is unknown. We demonstrate here that mutants deficient in the exopolysaccharide capsule have lowered intrinsic resistance to peptide antibiotics, while a mutation affecting sugar precursors involved in both capsule and lipopolysaccharide synthesis sensitizes the bacterium to multiple antibiotic classes. We observed that, when grown in the presence of certain antibiotics below their MIC, including the translation inhibitors chloramphenicol and erythromycin, *A. baumannii* increases production of the K locus exopolysaccharide. Hyperproduction of capsular exopolysaccharide is reversible and non-mutational, and occurs concomitantly with increased resistance to the inducing antibiotic that is independent of the presence of the K locus. Strikingly, antibiotic-enhanced capsular exopolysaccharide production confers increased resistance to killing by host complement and increases virulence in a mouse model of systemic infection. Finally, we show that augmented capsule production upon antibiotic exposure is facilitated by transcriptional increases in K locus gene expression that are dependent on a two-component regulatory system, *bfmRS*. These studies reveal that the synthesis of capsule, a major pathogenicity determinant, is regulated in response to antibiotic stress. Our data are consistent with a model in which gene expression changes triggered by ineffectual antibiotic treatment cause *A. baumannii* to transition between states of low and high virulence potential, which may contribute to the opportunistic nature of the pathogen.

## Author Summary

*Acinetobacter baumannii* has gained notoriety as a cause of hospital-acquired infections that are difficult to treat due to extensive antibiotic resistance. While the microorganism rarely causes disease in the community, it commonly infects patients receiving antibiotics. The factors intrinsic to the bacterium that enable growth in the presence of antibiotics are not well characterized. Furthermore, the consequences of subinhibitory antibiotic concentrations on *A*. *baumannii* disease are unknown. Here we examined the K locus, a bacterial disease determinant responsible for the production of protective surface polysaccharides, and asked whether this determinant also contributes to antibiotic resistance. We found that K locus polysaccharides facilitate resistance to multiple antibiotics, and, unexpectedly, that the bacterium responds to certain antibiotics at subinhibitory concentrations by increasing production of capsule, the principal K locus polysaccharide. This augmented production of capsule, which is mediated by upregulation of K locus gene expression, increased the ability of the bacterium to overcome attack by the complement system, an important anti-pathogen host defense, and result in lethal disease during experimental bloodstream infection in mice. Our studies indicate that *A*. *baumannii* increases its disease-causing potential in the setting of inadequate antibiotic treatment, which may promote the development of opportunistic infections.

## Introduction

Hospital-acquired infections with multidrug resistant (MDR) bacteria pose increasingly difficult challenges for patient care. These diseases are often intransigent to initial empiric broad-spectrum antibiotic therapy, delaying effective treatment and resulting in significant morbidity and mortality in already vulnerable patient populations. An emerging cause of such troublesome infections is *Acinetobacter baumannii*. This organism is responsible for a spectrum of diseases in susceptible individuals, including hospital-acquired pneumonia, sepsis, and wound infections [1]. *A*. *baumannii* is especially problematic in intensive care units (ICUs), where it is now among the 5 most common pathogens associated with ventilator-associated pneumonia in US hospitals [2–5]. Moreover, these infections are associated with alarming increases in drug resistance rates. A recent survey reported that most hospital-acquired *A*. *baumannii* infections are MDR [5], and strains resistant to all clinically useful antibiotics are emerging [6–8]. Observations such as these have led the Infectious Diseases Society of America and Food and Drug Administration to designate *A*. *baumannii* a high priority target for new antibiotic development [9,10]. Novel approaches to treat *A*. *baumannii* are urgently needed.

An understanding of the pathobiology of *A*. *baumannii* infections would facilitate the development of novel control strategies. These infections typically target critically ill, hospitalized patients with indwelling devices [1], in whom they can be found as colonizers before the onset of disease [11,12]. In addition, multivariate analyses have consistently identified prior or inappropriate antibiotic treatment as an independent risk factor for *A*. *baumannii* nosocomial diseases [13–15]. How antibiotics modulate host susceptibility to infection is not well understood, although several mechanisms are possible, including indirect effects on the host, such as reduction in competitive, drug-susceptible populations within the patient microbiota and/or modulation of innate immune defenses, as well as direct effects on *A*. *baumannii* physiology and virulence potential.

Regarding bacterial factors that contribute to pathogenicity, the *A*. *baumannii* envelope is associated with many of the determinants of virulence in mammalian disease models [16–22]. Among these, capsular exopolysaccharide has emerged as a universal virulence factor owing to several observations. In a study of greater than 40 *A*. *baumannii* patient isolates, almost all expressed a surface capsule [23]. Two recent bioinformatics studies of the genomes from a large number of clinical isolates have identified a sequence-variable gene cluster (the K locus) with predicted capsule biosynthesis functions [24,25]. Experiments with mutants deficient in certain of these functions, including polymer assembly and export [20] or subunit biosynthesis [17], have demonstrated roles for *A*. *baumannii* capsular exopolysaccharide in growth within soft tissue infection sites [20], lethality in a mouse septicemia model [17], defense against serum killing [17,20], and biofilm modulation [17]. Because of its importance in animal infection models and its immugenicity, the capsule has been proposed to be a target for protective antibody-based interventions [26].

How the complex surface polysaccharides determined by the K locus contribute to intrinsic antibiotic resistance is poorly characterized. The high intrinsic resistance of *A*. *baumannii* and related Gram-negative organisms such as the *Pseudomonads* is largely due to a cell envelope consisting of lipopolysaccharide (LPS) with low permeability to hydrophobic agents [27], “slow” porins with low permeability [28], and multiple drug efflux pumps [29]. Although not previously explored with *A*. *baumannii*, recent evidence supports a role for capsule structures in contributing to microbial defenses against macromolecular antibiotics such as antimicrobial peptides [30–35]. The *A*. *baumannii* outer envelope structures including capsule may lie at the interface of pathogenesis and drug resistance.

Here, we have examined the *A*. *baumannii* K locus with a view toward elucidating its contributions to the opportunistic nature of the bacterium. We determined how the capsular exopolysaccharide and major LPS glycoform defined by the K locus facilitate resistance to antibiotic treatment, and how antibiotics at doses insufficient to block bacterial replication cause regulatory changes in K locus expression that result in increased capsule production. We show, moreover, that this hyperproduction augments the ability of the organism to survive in the presence of host complement and cause virulent infections in mice. By uncovering conditions in which the protective exopolysaccharide capsule is hyperproduced, these studies have implications for the pathogenesis of *A*. *baumannii* infections in the antibiotic-treated patient.

## Results

### Deletions within the K locus reveal requirement of the Wzc tyrosine kinase for viability in rich media

**Fig 1:**
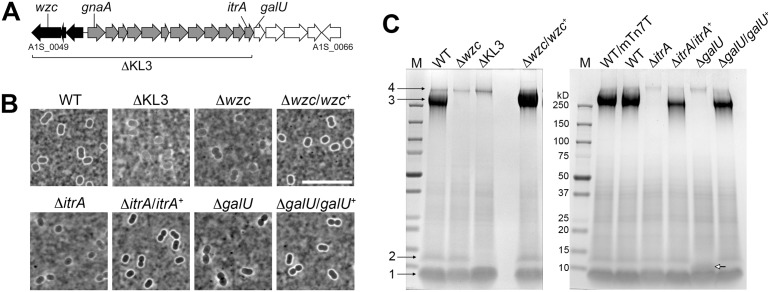
*A*. *baumannii* K locus genes determine major surface polysaccharides, including capsular exopolysaccharide and an LPS glycoform. **A**. The K locus from *A*. *baumannii* strain 17978 (KL3 in [25]) with genes analyzed in this study indicated. Black, gray, and white shading indicate distinct modules described in the text. **B**. India ink staining of bacteria grown to early post-exponential phase. Scale bar: 10μm. **C**. Analysis of polysaccharides in early post-exponential phase cell lysates separated by SDS-PAGE and stained with alcian blue. Numbered black arrows and white arrow indicate the principal polysaccharides described in the text. M, BioRad prestained MW marker. WT/mTn7T denotes the presence of the miniTn7 insertion without cloned gene.

The organization of the *A*. *baumannii* K locus responsible for capsule production is shown in Fig. 1A. While the gene composition of the K locus can exhibit substantial diversity across isolates, it generally encompasses a modular architecture including two highly conserved flanking units. At the 5’ end of the cluster, a highly conserved capsular polysaccharide assembly and export unit forms one universal module (Fig. 1A black arrows). This module includes *wzc* (A1S_0049, also referred to as *ptk*), having an *E*. *coli* ortholog that encodes a critical component of the export machinery controlling high-order Group 1 capsule assembly [36]. Control of capsule assembly by Wzc and its orthologs depends on a critical N-terminal tyrosine kinase domain [37]. A transposon insertion in *wzc* of *A*. *baumannii* strain 307–0294 completely abrogates capsular exopolysaccharide production [20]. A second highly conserved module composed of simple UDP-sugar synthesis genes lies at the 3’ end of the cluster (Fig. 1A, white arrows). The intervening region (Fig. 1A, gray arrows) contains several genes specific to each capsule type but invariably contains an initiating glycosyltransferase *itrA* (A1S_0061, also referred to as *pglC*) necessary to construct the glycan repeat-units that make up the high-order capsular exopolysaccharide as well as O-linked protein glycans [17,25].

In order to probe the functions of the complex polysaccharides determined by the *A*. *baumannii* K locus, we constructed deletions of key K locus genes in reference strain 17978 via homologous recombination. We first constructed strains harboring a deletion of *wzc*. After repeated attempts, we were able to isolate four such deletion mutants, each of which had the rough colony morphology expected for acapsular bacteria. Consistent with the model that these strains harbor second-site mutations required for viability, three of the strains had delays in growth kinetics compared to the WT strain (S1A Fig; isolates 16, 17 and 27) that were not rectified after replacement with the WT *wzc* allele (S1B Fig), although the colonies had a WT-appearing, smooth colony morphology. In the case of the fourth strain (isolate 9), when a WT *wzc* allele was re-introduced, a highly mucoid phenotype resulted, and we mapped the causative point mutation to *bfmS* (A1S_0749). The *bfmS* mutant generally suppressed the growth defect caused by the absence of *wzc*, as we could easily isolate ∆*wzc* strains in the *bfmS* mutant background. Further analysis of *bfmS* and its effects on capsule synthesis is presented in a subsequent section of this work. We conclude that loss of *wzc* in the 17978 background is likely to be lethal in the absence of suppressor mutations. ∆*wzc* isolates 16 and 27, for which reintroduction of *wzc* resulted in WT colony morphology, were utilized in the analyses that follow.

Decreased viability with mutations in complex polysaccharide synthesis pathways in other systems is thought to involve accumulation of toxic intermediates or the sequestration of essential lipid carriers [38]. We therefore generated additional deletions that would be predicted to bypass these lethal processes. We constructed a deletion, termed ∆KL3, that eliminates both the capsule export module as well as biosynthesis genes of the K locus (A1S_0049 through A1S_0061, see Fig. 1A)[17,25]. Mutants were also constructed by deleting the initiating glycosyltransferase *itrA*, or *galU* (A1S_0062), the first gene of the simple UDP-sugar synthesis module that encodes a predicted UTP-glucose-1-phosphate uridylyltransferase [24,25]. Multiple independent isolates of these mutants, each of which exhibited the expected rough colony morphology, were easily obtained, and the isolates had growth kinetics that matched that of WT (S1C, D Fig).

### K locus genes determine the principal *A*. *baumannii* surface polysaccharides

Like most *A*. *baumannii* isolates, strain 17978 displays a thin capsule (Fig. 1B, WT), and we assessed the various K locus mutants using India ink staining. The K locus mutants lacked the smooth, uniform capsule of the WT and were instead associated with a patchy, thinner outline (Fig. 1B). Reintroduction of the corresponding WT allele in the chromosome of the ∆*wzc*, ∆*itrA*, and ∆*galU* mutants restored the WT-appearing, smooth capsular halo (Fig. 1B). We next examined the polysaccharides produced by each strain after fractionation of cell lysates by SDS-PAGE and staining with the polysaccharide-specific dye, alcian blue (see Materials and Methods)[39,40]. With the WT strain, 4 major bands were detected (Fig. 1C). Band 3 depends on the genes of the K locus (Fig. 1C and S1E Fig) and has a migration pattern consistent with capsular exopolysaccharide [17]. Bands 1 and 2 co-migrate with LPS [41,42], with band 1 consistent with truncated, deep rough glycoforms of LPS [19]. Band 2 is consistent with LPS containing substituents that require the function of K locus proteins (Fig. 1C and S1E Fig). The *galU* deletion alters the migration of band 2 (Fig. 1C white arrow), consistent with a partially truncated LPS intermediate. Band 4 represents an additional high-molecular weight (MW) polysaccharide that is not dependent on the K locus, which may be poly-N-acetyl-glucosamine (PNAG) [43].

### The activity of the tyrosine kinase Wzc is required to maintain regulation of capsular exopolysaccharide assembly

**Fig 2:**
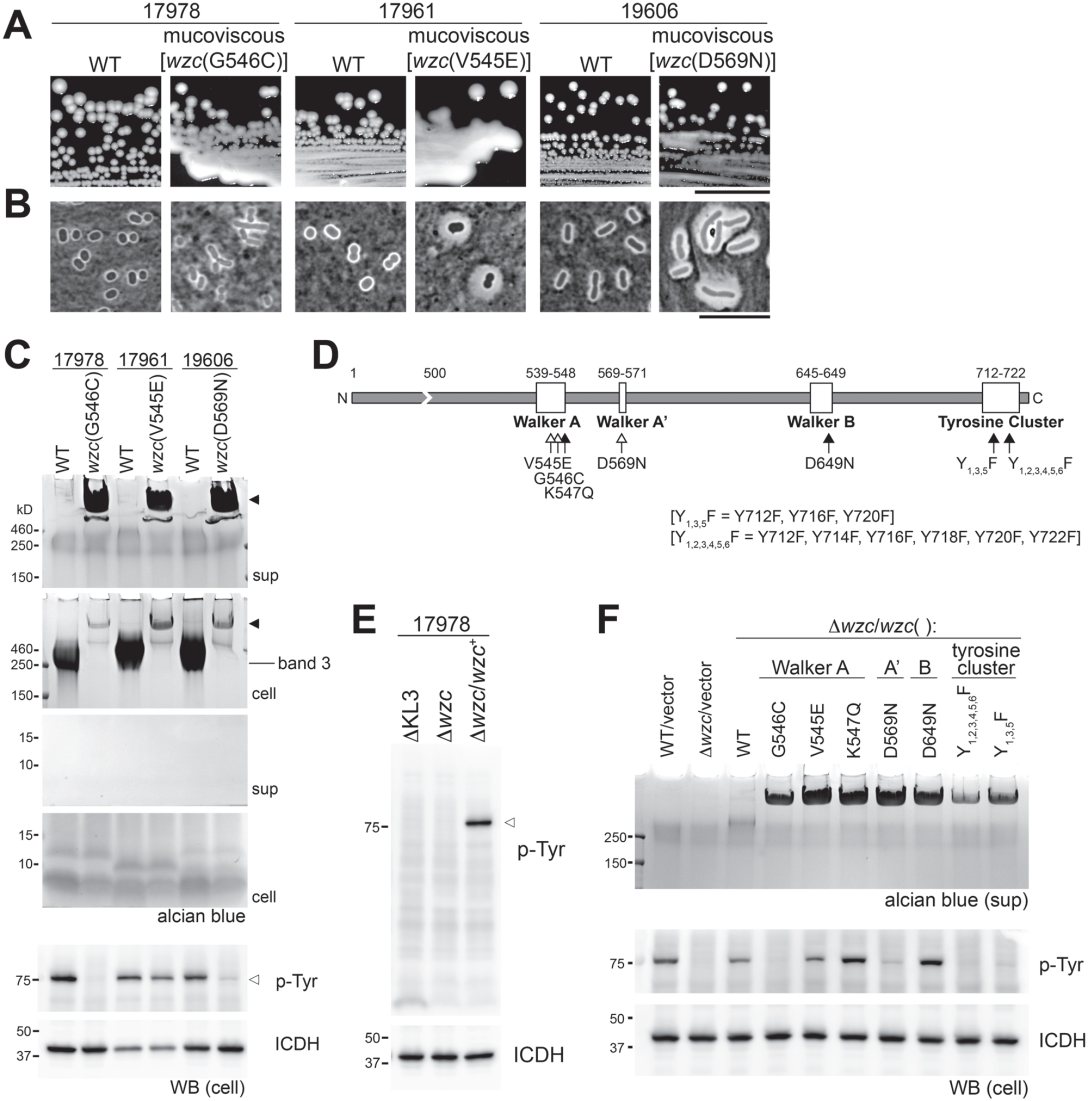
*A*. *baumannii* Wzc autokinase determines capsular exopolysaccharide polymer length. **A-D**. Spontaneous point mutations in autokinase domain result in misregulation of capsular exopolysaccharide production. **A**. Colony morphology on LB plates. Scale bar: 1cm. **B**. Capsules were analyzed by India ink staining (Scale bar: 10μm). **C**. Polysaccharides were analyzed by alcian blue staining of cell lysates and culture supernatants fractionated by SDS-PAGE; filled arrowheads denote misregulated, very high-MW capsular exopolysaccharide. The location of WT capsular exopolysaccharide corresponding to band 3 in Fig. 1C is noted. Lysates were also blotted and stained with 4G10 antibodies to probe for Wzc-dependent phosphotyrosines (open arrowhead), and with anti-ICDH antisera as loading control. **D**. Schematic of the Wzc tyrosine kinase domain indicating the location of point mutations relative to conserved motifs. Open arrows indicate spontaneous mutations, and filled arrows indicate engineered mutations. **E**. Lysates were immunoblotted as in C. **F**. *wzc* alleles with point mutations in conserved tyrosine kinase motifs were reconstituted in a ∆*wzc* strain. Culture supernatants were fractionated and stained with alcian blue. Lysates were blotted and probed as above.

Stocks of commonly studied *A*. *baumannii* strains (17978, 19606, and 17961) generate mucoviscous colony variants at a high frequency that alter band 3 content (Fig. 2A and C). These mutants are associated with viscous, sticky strings when lifted with a toothpick. Two of the three mutants displayed capsules with increased thickness upon inspection with India ink (Fig. 2B), and lysates of all three mutants contained a very high MW polysaccharide (Fig. 2C, “cell” panel, filled arrowhead) that replaced the band 3 capsular polysaccharide of WT. The majority of this polysaccharide was found in culture supernatants (Fig. 2D, “sup” panel, filled arrowhead). Genome sequencing indicates that each mucoviscous variant has a SNP in the *wzc* regulatory autokinase domain (Fig. 2D, white arrows). To test the model that these mutations alter Wzc activity, we probed the *A*. *baumannii* lysates with anti-phosphotyrosine. We note that, when a phosphotyrosine signal was detected in the resulting western blots, only a single band was present; this band depended on Wzc and matched its size (Fig. 2E, open arrowhead), indicating that it represents Wzc autophosphorylation. Consistent with the mutational changes altering Wzc activity, the mucoviscous mutants have lowered autophosphorylation levels compared to the WT strain (Fig. 2C, open arrowhead). Expressing the *wzc* point mutants on a plasmid in a ∆*wzc* background reconstituted the production of loosely associated, very high MW polysaccharide (Fig. 2F). These results indicate that autophosphorylation of Wzc negatively regulates capsule polymer chain length.

Engineered mutations of key Walker Box residues (K547, D649) or the C-terminal phosphorylation site tyrosines (Fig. 2D, black arrows) in Wzc support the model that the autokinase activity negatively regulates capsule production. Each of these alleles caused very high MW polysaccharide production (Fig. 2F), although autophosphorylation was detected in some of the engineered Walker box mutants (Fig. 2F). Together these data demonstrate that the principal *A*. *baumannii* exopolysaccharide is a high MW capsule whose assembly is controlled by Wzc.

### K locus polysaccharides facilitate intrinsic antibiotic resistance

**Fig 3:**
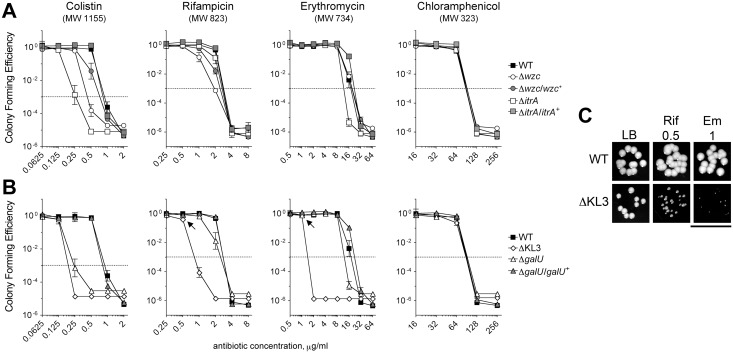
*A*. *baumannii* K-locus polysaccharides facilitate intrinsic antibiotic resistance. **A, B.** Colony forming efficiency (CFE) tests with WT and mutant *A*. *baumannii*. Bacteria were serially diluted, plated onto LB agar or LB agar containing the indicated antibiotics, and the resulting CFU were scored after overnight incubation at 37°C. The limit of detection was approximately 10^–6^, except for assays with colistin in which the limit of detection was approximately 10^–5^. MIC (Table 1) is defined as the antibiotic concentration at which CFE drops below 10^–3^ (dotted line). Data points represent the mean ± SEM from at least two independent cultures. Where not visible, error bars are within the confines of the symbol. The MW of each antibiotic is indicated. **A**. Mutants deficient in capsular exopolysaccharide were tested; the ∆*wzc* isolate tested was isolate 16. **B**. Mutants deficient in capsular exopolysaccharide and LPS were tested. **C**. At concentrations resulting in ~100% CFE (arrows in **B**), Em and Rif reduce the growth of ∆KL3 but not WT bacteria. Scale bar: 1cm.

We next interrogated the roles of K locus polysaccharides in defense against antibiotics using the deletion mutants with specific deficiencies in these structures. Studies with diverse pathogens have shown that a polysaccharide capsule can confer resistance to antimicrobial peptides [30–35] and other large antibiotics [32]. We tested whether mutants deficient in the *A*. *baumannii* capsular exopolysaccharide have increased sensitivity to the antimicrobial peptide colistin (Col), a critical last-line antibiotic for MDR *A*. *baumannii*, as well as the bulky, hydrophobic antibiotics erythromycin (Em) and rifampicin (Rif). To quantitate antibiotic resistance, we enumerated the growth of bacterial populations on agar containing serial dilutions of each antibiotic. With this assay, the ∆*wzc* and ∆*itrA* mutants, which are completely deficient in capsule but have an intact LPS, consistently displayed increased sensitivity to Col, and reintroduction of the respective WT allele restored resistance to near WT-levels (Fig. 3A and Table 1). Compared with Col, resistances to Rif and Em were affected to a lesser extent and less consistently across the mutants (Fig. 3A and Table 1). Resistance to the small antibiotic chloramphenicol (Cm) was unaffected (Fig. 3A and Table 1). We note that because the ∆*itrA* mutant is impaired in both capsule production and O-linked protein glycosylation [17], we cannot exclude the possibility that a loss of protein glycans contributed to the phenotype of decreased antibiotic resistance observed with this strain.

**Table 1:** Minimal Inhibitory Concentrations (MICs) determined from antibiotic resistance assays (Fig. 3).

	MIC (μg/ml)				
		Col	Rif	Em	Cm
**WT**		1	4	32	128
**∆*wzc***		0.5	2	32	128
**∆*wzc*/*wzc*** ^**+**^		1	4	32	128
**∆*itrA***		0.5	4	16	128
**∆*itrA*/*itrA*** ^**+**^		1	4	32	128
**∆*galU***		0.25	4	16	128
**∆*galU*/*galU*** ^**+**^		1	4	32	128
**∆KL3**		0.25	1	2	128

We next tested the mutants altered in production of both the exopolysaccharide capsule and the K locus-dependent LPS glycoform. Similar to the strains deficient in only capsule, the ∆*galU* mutant, which is deficient in capsule and expresses a partially truncated LPS, showed increased sensitivity to Col, along with mild increases in sensitivity to Rif and Em; these phenotypes reverted to WT upon reintroduction of the *galU* gene (Fig. 3B and Table 1). By contrast, the ∆KL3 mutant demonstrated a large increase in sensitivity to Col, Rif, and Em (Fig. 3B and Table 1), consistent with the phenotypes of Gram-negative mutants lacking core LPS sugars [16,44,45]. Moreover, at concentrations of Em and Rif that permitted 100% colony forming efficiency (CFE) with ∆KL3 (Fig. 3B, arrows), growth inhibition was observed (Fig. 3C). Mutants with altered envelope permeability due to LPS defects usually are not hypersusceptible to small, hydrophilic antibiotics, which generally enter the cell though porin channels [46]. Consistent with this idea, ∆KL3 had no increased susceptibility to the small antibiotic Cm (Fig. 3B and Table 1). These data demonstrate that the K locus polysaccharides facilitate intrinsic resistance to multiple classes of antibiotics, with the exopolysaccharide capsule contributing principally to defense against antimicrobial peptides.

### Sub-MIC translation-inhibitor antibiotics induce hyper-production of capsular exopolysaccharide by rapid, reversible, and non-mutational means

**Fig 4:**
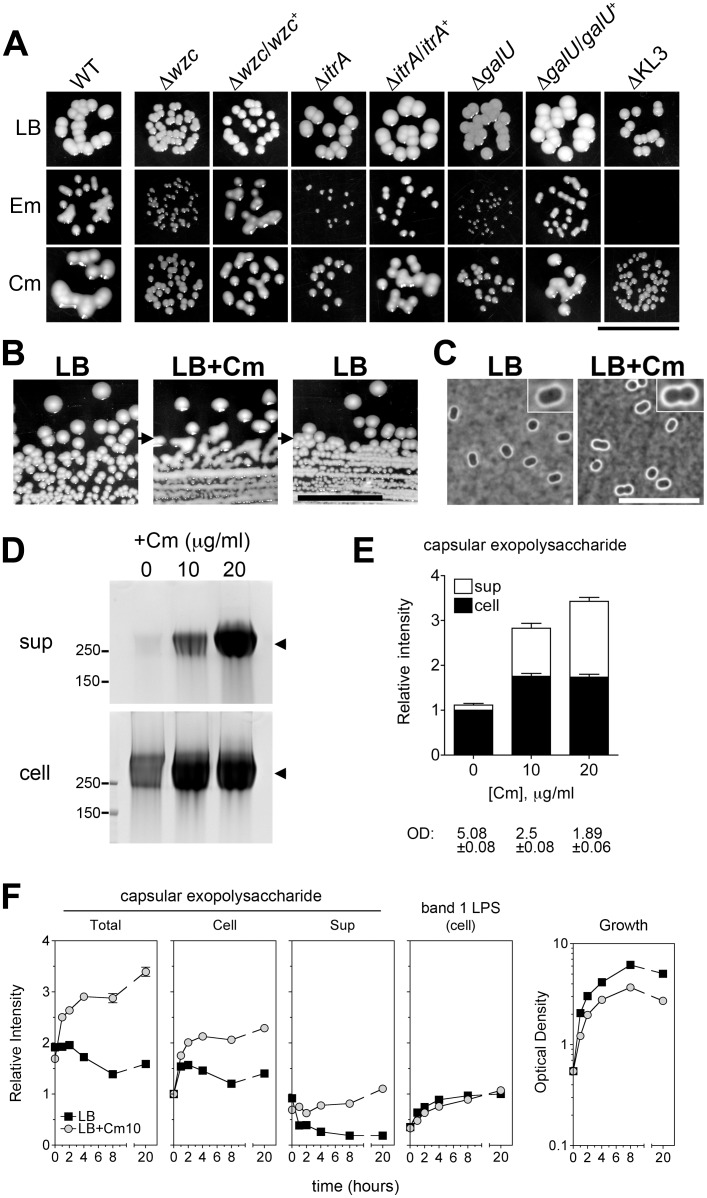
Rapid and reversible induction of capsular exopolysaccharide by translation-inhibitor antibiotics. **A**. At concentrations below their MIC and yielding ~100% CFE, Em and Cm cause mucoid colony morphology dependent on the K locus genes. Colonies grown on LB plates with 4 μg/ml Em, 32–64 μg/ml Cm, or no antibiotic were imaged after 1 day at 37°C followed by overnight at room temperature. With ∆KL3 on the Em plates, no colonies were observed (see Fig. 3B). Scale bar: 1cm. **B**. The mucoid phenotype is lost upon restreaking onto plates lacking antibiotics. A colony of *A*. *baumannii* cells not previously exposed to antibiotics was picked from an LB agar plate and streaked onto LB agar containing 25 μg/ml Cm, resulting in mucoid growth. A colony from the Cm plate was then restreaked onto LB agar without antibiotics. Scale bar: 1cm. **C**. 0 or 10 μg/ml Cm were added to log phase bacteria and cells were stained with India ink after 4 hours. Images were acquired with identical exposure settings and are representative of three independent experiments. Scale bar: 10μm. Insets show enlarged views of representative bacteria. **D**. Cm was added to log phase bacteria at the indicated concentration, and lysates and cell-free supernatants collected after overnight incubation were analyzed with alcian blue. **E**. Densitometry of alcian-blue stained capsular exopolysaccharide (bands indicated by arrowheads in **D**). Culture optical density (OD) at time of sample collection is indicated. **F**. Densitometry of capsular exopolysaccharide (as in **E**) and K-independent LPS glycoform (corresponding to band 1 in Fig. 1C) in lysates collected over multiple time-points after Cm10 treatment. Data are presented as mean intensity ± SEM from three independent experiments. Where not visible, error bars are within the confines of the symbol.

While performing antibiotic sensitivity experiments we made the unexpected observation that growth on Cm and Em causes *A*. *baumannii* to assume a hypermucoid state (Fig. 4A). Hypermucoidy was not seen with Rif or Col, but was seen with Cm and Em across many clinical *A*. *baumannii* isolates. The colonies were highly mucoid but were not associated with viscous strings when picked, indicating a phenotype distinct from the mucoviscous Wzc kinase mutants above. Hypermucoidy occurred over a wide range of antibiotic concentrations at sub-MIC including concentrations that that showed no loss of CFE (Em, 2–16 μg/ml, and Cm, 8–64 μg/ml; see Fig. 3), suggesting that the mucoid colonies were not mutants. The mucoid phenotype is lost upon restreaking onto plates lacking antibiotics, and reappears upon restreaking onto Cm plates (Fig. 4B); this holds true after ten such passages, further supporting that the phenomenon is non-mutational. Hypermucoidy was dependent on the K locus genes and the ability to produce capsular exopolysaccharide, because the K locus deletion mutants prevented this phenotype, and reintroduction of the respective WT genes restored it (Fig. 4A).

To begin to analyze the connection between antibiotic exposure and exopolysaccharide production, we first examined the effects of sub-MIC antibiotics on polysaccharide levels. Using Cm as prototype, we treated logarithmically growing cells with concentrations (10 and 20 μg/ml) that are below the MIC (128 μg/ml; see Fig. 3) but that inhibit growth in liquid culture by about 50 and 65%, respectively (Figs. 4E and 4F). Cells challenged with Cm at 10 μg/ml displayed slightly thickened capsules as assessed by India ink (Fig. 4C). Furthermore, both cell-associated and cell-free capsular exopolysaccharide were increased approximately two- to three-fold based on SDS-PAGE fractionation (Fig. 4D and 4E). Of note, the migration pattern and apparent MW of the capsular exopolysaccharide was identical with untreated cells, indicating that polymer length was unchanged (Fig. 4D). The induction by Cm of capsular exopolysaccharide was dependent on the K locus genes (S2A Fig), and increased production of K locus-independent polysaccharides was not observed.

We next determined the kinetics of capsular exopolysaccharide induction in broth culture by Cm by analyzing fractionated culture lysates and supernatants over multiple post-treatment time points. With untreated cultures, cell-associated capsular exopolysaccharide levels generally peaked upon entry into post-exponential phase and then steadily declined (Fig. 4F, black squares, and S2B Fig). In contrast, cultures treated with Cm resulted in increased capsular polysaccharides as early as one hour after drug addition with continued accumulation over time (Fig. 4F, gray squares, and S2B Fig). Cell-associated LPS levels, however, appeared unaffected by Cm treatment (Fig. 4F and S2B Fig). These results indicate that Cm treatment induces a rapid and specific increase in capsular exopolysaccharide content.

**Fig 5:**
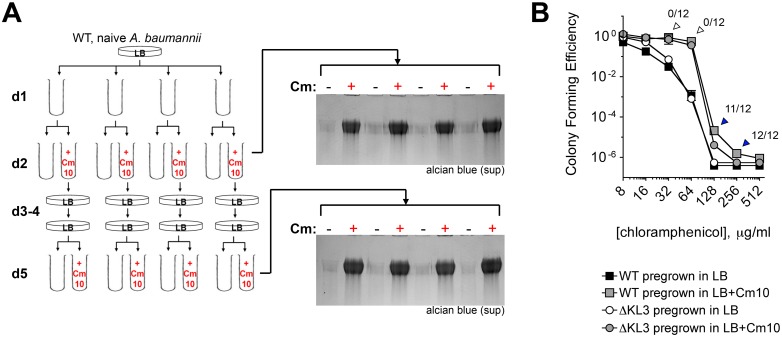
Induction of capsular exopolysaccharide by translation-inhibitor antibiotics is non-mutational. **A**. Sub-MIC Cm treatment does not select for mutants that constitutively hyperproduce exopolysaccharide. 4 independent lineages of WT bacteria not previously exposed to antibiotics were grown overnight with Cm10 (day 2) followed by restreaking in the absence of antibiotics (days 3–4). Colonies on day 4 were reinoculated into LB with or without Cm10. Capsular exopolysaccharide in cell-free supernatants was analyzed with alcian blue. **B**. Sub-MIC Cm pretreatment increases phenotypic resistance to Cm independent of the K locus. Cell populations from d2 were analyzed for CFE on Cm plates. Data are presented as in Fig. 3. Colonies arising from Cm10-pretreated WT cells plated on the indicated Cm concentration (arrowheads) were tested for mutational high-level Cm^R^ by passaging twice on LB agar without antibiotics, then scoring for increased growth on 100 μg/ml Cm. Fractions above the arrowheads denote the ratio of clones demonstrating increased growth on Cm compared to the WT grown in absence of Cm.

The reversible plating phenotype and rapid kinetics of capsular exopolysaccharide induction with drug treatment support the idea that this phenomenon is regulatory and non-mutational. To understand the extent to which mutant subpopulations may be involved in the exopolysaccharide hyperproduction phenomenon, we performed two additional tests. First, we determined if mutants constitutively hyperproducing capsular exopolysaccharide were selected after drug treatment under the conditions used in Fig. 4D-F by analyzing multiple lineages treated with drugs. As diagrammed in Fig. 5A, 4 independent lineages of WT *A*. *baumannii* not previously exposed to antibiotics were grown with Cm for 20h and then passaged twice on solid media lacking antibiotics. Cells were then reinoculated into liquid broth and again grown with or without Cm. Supernatants from cultures before (d2) and after (d5) passage on solid media without antibiotics were analyzed for differences in the levels of basal and drug-induced cell-free capsular exopolysaccharides to discern whether drug treatment selected for constitutive exopolysaccharide hyperproducers. As seen in Fig. 5A, the levels of capsular exopolysaccharide in the d5 cultures not treated with Cm were identical to those at d2, as were the levels of induced capsular exopolysaccharide in the Cm-treated cultures. In addition, all colonies appearing during passage on media without antibiotics on d3–4 were observed to be WT-appearing and non-mucoid. These results indicate that the phenotypic increase in capsular exopolysaccharide levels by Cm addition at sub-MIC was not the result of an outgrowth of exopolysaccharide hyperproducing mutants, and instead was due to a highly reversible, population-wide regulatory response.

Second, to determine if subpopulations of mutationally drug-resistant clones were selected, bacteria from d2 (Fig. 5A) were also analyzed for high-level resistance to Cm by quantifying the number of bacteria able to form colonies at increasing concentrations of drug. Pretreatment with sub-MIC Cm (Fig. 5B, gray squares) resulted in an increase in the proportion of the population able to grow on Cm at 32–256 μg/ml compared to cells pregrown without the antibiotic (Fig. 5B, black squares). A similar effect was observed with ∆KL3 bacteria, indicating that the increased resistance was independent of the K locus polysaccharides (Fig. 5B, circles). The colonies arising after plating Cm-pretreated WT *A*. *baumannii* on Cm at 32–256 μg/ml (arrowheads) were then tested for mutational resistance at 100 μg/ml Cm. The increased growth on 32–64 μg/ml Cm was not mutational, because all clones isolated from these concentrations behaved as the parental WT when retested for high-level Cm resistance at 100 μg/ml Cm (open arrowheads). This phenotypic increase in CFE by sub-MIC Cm may be related to a number of possible mechanisms, one of which may be upregulation of efflux activities as shown with *P*. *aeruginosa* [47]. By contrast, the increased growth on 128–256 μg/ml Cm, which is above the MIC, was mutational because most or all of the clones at these concentrations had high-level Cm resistance when retested for growth on Cm100 plates (blue arrowheads). These data indicate that exposure to sub-MIC Cm stimulates multiple bacterial responses including increased capsule production and induction of K locus-independent resistance mechanisms. Furthermore, these responses are largely non-mutational.

### Induction of capsular exopolysaccharides increases resistance to complement killing *ex vivo* and augments virulence during systemic infection

Augmented capsular exopolysaccharide expression in response to environmental triggers has implications for multiple facets of disease with *A*. *baumannii*. We first asked whether capsule hyperproduction increases resistance to the peptide antibiotic Col, to which, unlike Cm, full intrinsic resistance depends on the presence of capsular exopolysaccharide. Increasing capsule production by transient pre-treatment with sub-MIC Cm, however, conferred no additional protection from the bactericidal activity of Col (S3 Fig).

**Fig 6:**
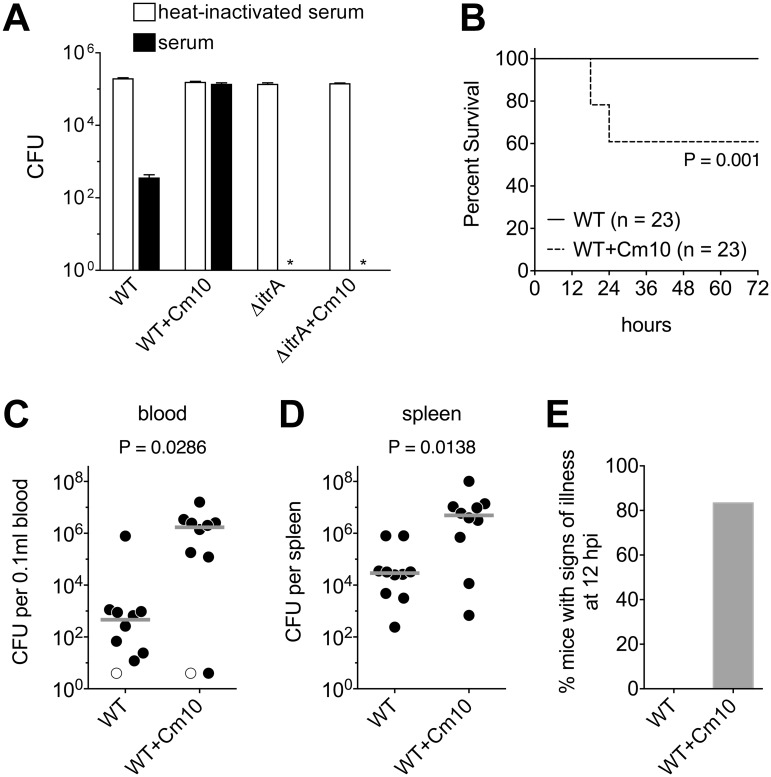
Antibiotic-induced exopolysaccharide hyperproduction increases serum resistance and virulence. **A**. Approximately 10^5^ WT and ∆*itrA A*. *baumannii* cells grown transiently in the absence or presence of sub-MIC Cm (10 μg/ml) were incubated with baby rabbit serum (black bars) or heat-inactivated baby rabbit serum (white bars) for 1 hour, then serially diluted and plated on LB agar without antibiotics to determine viable counts. Data are presented as mean ± SEM from four assays. The asterisks indicate that viable ∆*itrA* bacteria were not detected after incubation with active serum. Comparison of bacterial counts at the start and end of incubation with heat-inactivated serum indicated that all strains replicated approximately once during the course of the assay. **B**. Survival of mice infected intraperitoneally with WT *A*. *baumannii* grown transiently in the absence or presence of sub-MIC Cm (10 μg/ml). Data are pooled from three independent experiments with 7–8 mice per group in each experiment. Statistical significance was determined by the Gehan-Breslow-Wilcoxon Test. Mean CFU in the inocula were 1.4x10^8^ (untreated) and 1.1x10^8^ (Cm10 treated). **C-E**. Mice were infected as above and were euthanized after 12 hours. Mean CFU in the inocula were 1.3x10^8^ (untreated) and 7.9x10^7^ (Cm10 treated). Bacterial burdens in the blood (**C**) and spleen (**D**) were determined. Each symbol represents one animal, with open symbols denoting values below the limit of detection. Gray lines are median values. Data are pooled from two independent experiments with 5 mice per group in each experiment. Statistical significance was determined by the Mann-Whitney test. Mice were scored for the presence of signs of illness (hunched posture, suppressed activity, and ruffled fur) (**E**); data include all mice analyzed in C and D, plus two additional mice infected with Cm10-pre-treated bacteria from which blood and organ samples were not collected.

Because capsule determines evasion of killing by the complement system [17,20], an important host defense mechanism, we tested the hypothesis that capsule hyper-production upon antibiotic exposure can confer increased resistance to complement killing. *A*. *baumannii* cells were grown in the presence or absence of sub-MIC Cm and then incubated with rabbit serum as a source of complement, followed by serial dilution and plating onto medium without antibiotics to determine viable counts. In the absence of Cm pretreatment, incubation in serum resulted in 2–3 logs of killing with WT bacteria, and approximately 5 logs of killing with the ∆*itrA* mutant which lacks capsule but has an intact LPS (Fig. 6A). This killing was consistent with a previous study using human serum [17], and was entirely dependent on active complement components because incubation with heat-inactivated serum resulted in complete survival in all cases (Fig. 6A). Notably, pre-treatment of WT bacteria with sub-MIC Cm conferred increased survival compared to untreated cells, resulting in high-level serum resistance (Fig. 6A). The ability to acquire high-level serum resistance required capsular exopolysaccharide, because pre-treatment of the ∆*itrA* mutant with sub-MIC Cm did not result in a commensurate level of protection (Fig. 6A). These data demonstrate that treatment with an antibiotic at sub-MIC levels sufficient to augment capsule production increases resistance to the lethal effects of complement.

Based on this result, we predicted that antibiotic-treated bacteria would be more virulent during bloodstream infection. To evaluate virulence, bacteremia was established in mice by intraperitoneal injection of *A*. *baumannii* [48]. In experiments analyzing the survival of infected mice with this model, we found that a dose of approximately 10^8^ WT bacteria grown under standard laboratory conditions was unable to cause lethality (Fig. 6B), consistent with previous reports demonstrating the low virulence of strain 17978 [49,50]. When enhanced capsule production was induced by pre-treating bacteria with sub-MIC Cm, however, a significant decrease in host survival was observed at the same dose (Fig. 6B; P = 0.001). We hypothesized that this elevated virulence was the outcome of increased bacterial loads in the bloodstream and deep tissues of the host. To test this, we analyzed bacterial burdens in the blood and spleen of additional groups of mice infected for 12 hours. Consistent with the above hypothesis, antibiotic pre-treatment of bacteria resulted in a >3 log increase in bacterial counts in the bloodstream and a >2 log increase in bacterial counts in the spleen (Fig. 6C, D). Moreover, in these experiments, all mice infected with control bacteria appeared healthy, but the vast majority of mice infected with antibiotic pre-treated bacteria displayed signs of illness at the 12-hour endpoint (Fig. 6E). Together, these data demonstrate that exposure to antibiotics that enhance capsule production manifests in increased virulence during systemic infection.

### Sub-MIC Cm increases K locus gene expression at the transcriptional level

**Fig 7:**
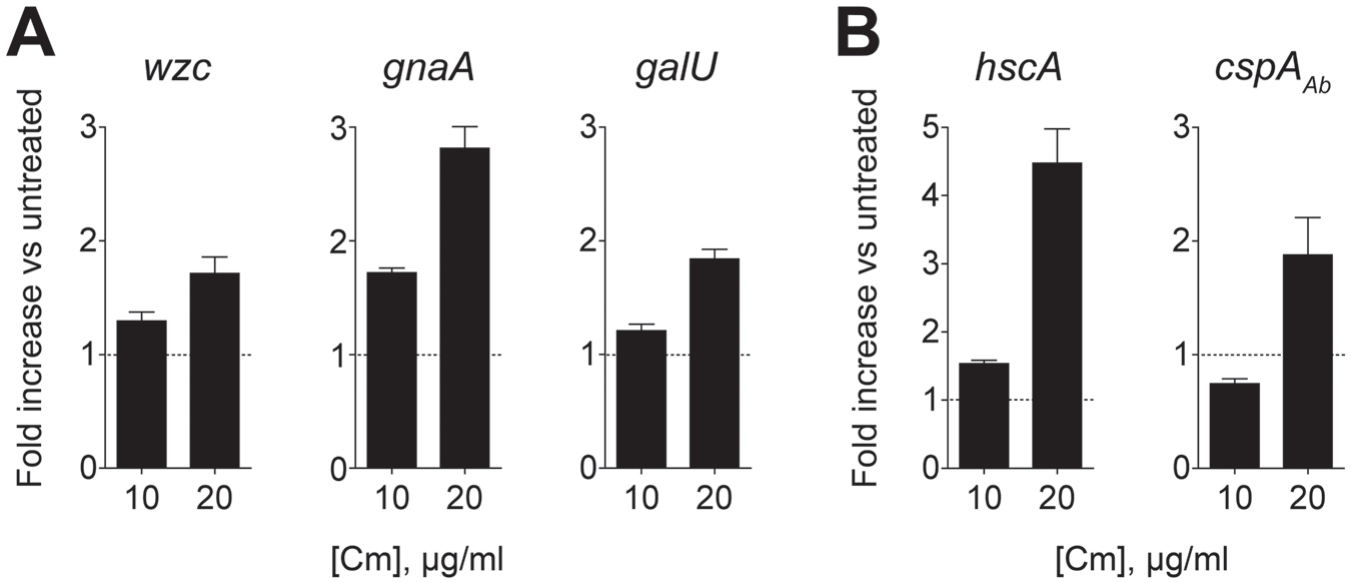
Capsular exopolysaccharide hyperproduction is associated with transcriptional increases in K locus and cold-shock gene expression. cDNA synthesized from RNA isolated 2h post-treatment with sub-MIC Cm was probed via qRT-PCR with primers specific for K locus (**A**) and cold-shock (**B**) genes. Fold change in transcript levels in treated vs untreated cells was determined, and data are presented as mean ± SEM from 8 independent cultures. P<0.01 in all cases as determined by one sample t test with a reference value of one.

To understand how induction of capsular polysaccharide production by antibiotics occurs, we analyzed the effects of sub-MIC Cm on K locus gene transcripts after 2 hours of drug exposure, focusing on 3 representative K locus genes critical for capsule synthesis: *wzc*; *galU*; and *gnaA*, the first gene in the unit divergently transcribed from the export module (see Fig. 1A) that encodes a UDP-D-GlcNAc to UDP-D-GlcNAcA dehydrogenase [24,25]. Compared to untreated cells, exposure to sub-MIC Cm resulted in significantly increased transcripts for each of these genes (Fig. 7A). The transcriptional increase was modest with 10 μg/ml Cm but commensurate with the increased levels of capsular exopolysaccharide detected at 2 hours with this condition (Fig. 4F). Since capsular exopolysaccharide is expected to accumulate with Cm treatment during post-exponential phase, these early increases in transcript levels are also in agreement with the overall increases in exopolysaccharide levels observed after prolonged exposure in post-exponential phase cultures (Fig. 4E).

We explored the possibility that exopolysaccharide hyper-production could additionally involve post-transcriptional changes in K locus gene expression. While Wzc appeared to be the principal protein modified by phosphotyrosine in cell lysates, we hypothesized that sub-MIC Cm may result in novel phosphotyrosine modifications that could contribute to enhanced capsule biosynthesis activities. Treatment with neither low nor high concentrations of Cm, however, resulted in any detectable novel phosphotyrosine signals after lysates were analyzed by western blotting (S4 Fig).

In *E*. *coli*, Cm and Em exposure induces changes in the transcription levels of cold shock genes [51–54]. This is in contrast to other protein synthesis inhibitors such as aminoglycosides that induce an opposing response virtually identical to that observed with heat-shock [53]. We have observed that the aminoglycosides gentamicin and streptomycin do not result in hypermucoid colony morphology or substantial increases in exopolysaccharide production, consistent with the model that antibiotics inducing cold shock are associated with enhanced exopolysaccharide synthesis. To test this model, we identified orthologs of the cold-shock genes *hscA* and *cspA* [52,55] in *A*. *baumannii* and probed their transcription levels after treatment with sub-MIC Cm. As shown in Fig. 7B, Cm at 20 μg/ml results in transcriptional induction of these genes, while treatment with 10 μg/ml results in an increase in only *hscA* transcript at the time point analyzed, consistent with cold shock gene induction coinciding with K locus gene induction.

### The BfmRS TCS and capsule hyperproduction

We noted that the hypermucoid phenotype assumed by WT cells growing with sub-MIC Cm or Em was highly similar to the hypermucoid plate phenotype that resulted when a WT *wzc* allele was re-introduced to ∆*wzc* isolate 9, which contains a suppressor mutation that mapped to *bfmS*. BfmS is the receptor histidine kinase (HK) component of *bfmRS*, a highly conserved TCS in *A*. *baumannii* initially identified as a locus critical for biofilm formation and production of pili [56], and shown to modulate porin localization [57]. In addition, results from two genome-wide screens have indicated a role for BfmRS in promoting *A*. *baumannii* survival within the mammalian host [22,58]. Because of the genetic interaction between *wzc* and *bfmS*, in which mutation of *bfmS* suppresses the toxicity of ∆*wzc*, and the highly similar plate phenotypes between a *bfmS* strain and sub-MIC antibiotic treatment, we sought to characterize how BfmRS affects K locus expression in response to antibiotics.

**Fig 8:**
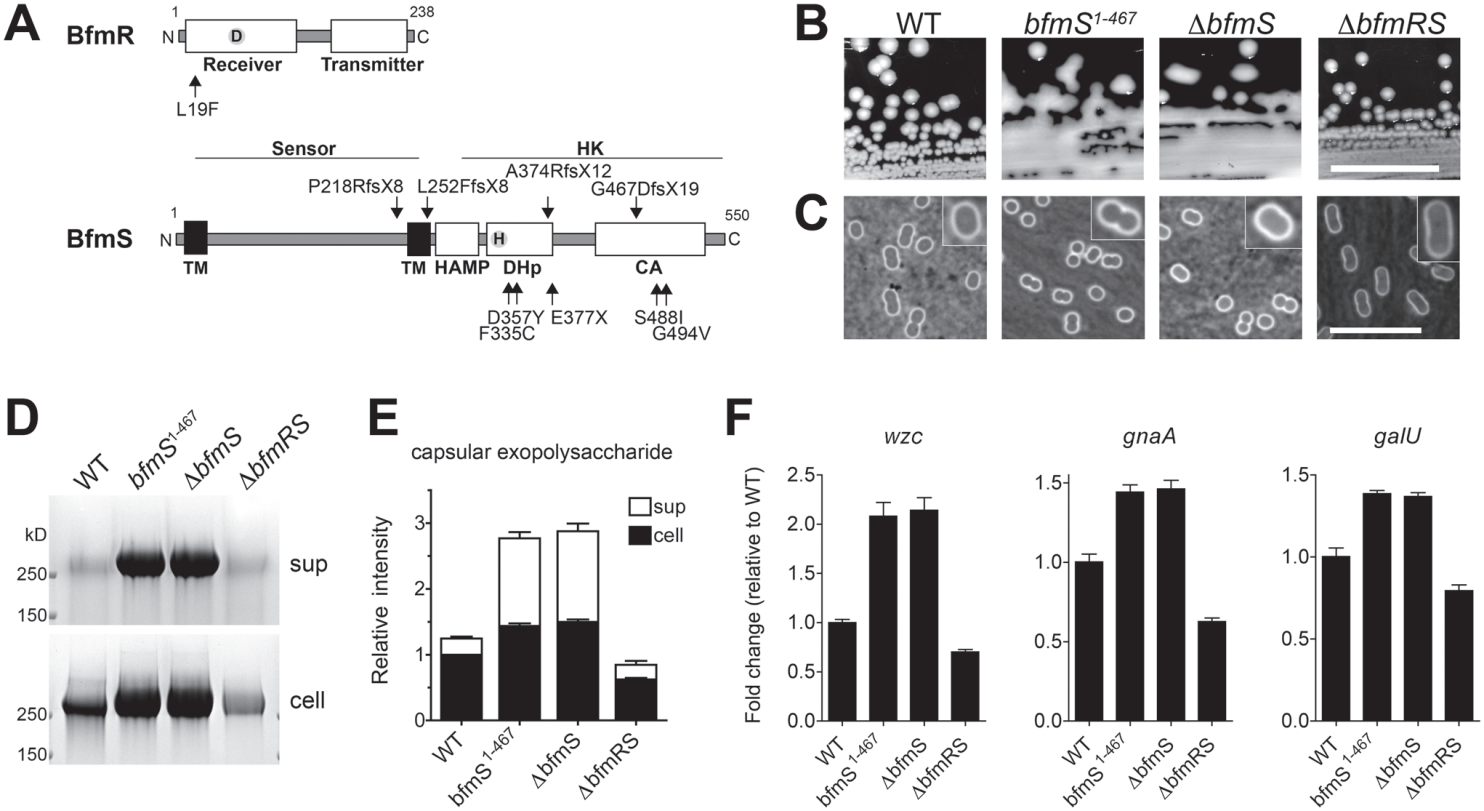
The *A*. *baumannii* BfmRS TCS controls capsule hyperproduction. **A**. Schematic of BfmR and BfmS indicating the location of mutations relative to conserved domains in bacterial response regulators and receptor HKs [85]. **B**. Colony morphology on LB agar plates. Scale bar: 1cm. **C-F**. Bacteria were grown to early post-exponential phase. Capsules were analyzed by India ink (**C**, Scale bar: 10μm). Consistent with a previous study, bacteria lacking *bfmR* had a more elongated cellular morphology [56]. Exopolysaccharides were analyzed by alcian blue staining (**D, E**) as in Fig. 4. **F**. Transcripts were examined via qRT-PCR as in Fig. 7 with data plotted as mean fold change relative to WT ± SEM from 3 independent cultures. P<0.01 in all cases comparing WT vs mutant, as determined by one-way ANOVA with Dunnett’s post test, except when comparing change in *wzc* between WT and ∆*bfmRS* (P>0.05).

A group of spontaneous hypermucoid mutants was isolated that had amino acid changes in the *bfmRS* locus (Table 2 and Materials and Methods). In BfmS, these mutations truncate the protein or are predicted to disrupt key motifs within the HK domain (Fig. 8A). In BfmR, the mutated residue (Fig. 8A) corresponds to the exact position in the close *E*. *coli* ortholog RstA required for phosphotransfer [59], with the result that the mutant is expected to be phosphorylation-negative. These observations all fit a model in which the BfmS HK negatively regulates capsule expression through phosphorylation of its cognate receiver. This model predicts that deletion of *bfmS* would lead to constitutive capsule hyperproduction, while deletion of *bfmR* or the complete two-gene operon would not.

**Table 2:** Spontaneous mutations in *bfmRS* resulting in hypermucoid phenotype.

gene	mutation	residue change(s)	domain affected
***bfmS*** ^a^	deletion of C_653_	P218RfsX8	sensor (truncation)
	tandem duplication of C_722_ to T_755_ (34bp)	L252FfsX8	TM2 (truncation)
	T1004G	F335C	DHp
	G1069T	D357Y	DHp
	ISAba11 insertion with duplication of A_1114_ to T_1119_	A374RfsX12	DHp (truncation)
	G1129T	E377X	DHp/CA (truncation)
	deletion of G_1400_	G467DfsX19	truncation
	G1463T	S488I	CA
	G1481T	G494V	CA (G2 box)
***bfmR***	A57T	L19F	receiver

^a.^ *bfmS* ORF starts with GTG upstream of annotated start site in GenBank (CP000521.1 888969..890618)

We tested this model by constructing the respective deletions and analyzing their effects on polysaccharide production. As predicted by the model, ∆*bfmS* resulted in a hypermucoid plate phenotype (Fig. 8B) and slightly thickened capsule (Fig. 8C), as did the truncation allele affecting the HK domain, *bfmS* ^1–467^. Deletion of *bfmS* in another *A*. *baumannii* strain, 19606, also resulted in a hypermucoid plate phenotype (S5A Fig). By contrast, ∆*bfmR* and ∆*bfmRS*, which showed identical phenotypes, resulted in rough colony morphologies with no appreciable increase in capsule by India ink staining (Fig. 8B, C; ∆*bfmR* shown in S5B, C Fig). Fractionation of polysaccharides by SDS-PAGE revealed that capsular exopolysaccharide levels were consistent with these phenotypes. Compared to WT, ∆*bfmS* and *bfmS* ^1–467^ resulted in increased cell-associated and cell-free capsular polysaccharides, while ∆*bfmRS* caused an overall decrease (Fig. 8D, E). These changes in polysaccharide abundance were due to transcriptional changes in K locus gene expression, as demonstrated by qRT-PCR analysis of *wzc*, *gnaA*, and *galU* gene transcripts (Fig. 8F). Together these results demonstrate that the BfmRS TCS sets the level of K locus gene expression, and are consistent with a model in which phosphorylation of BfmR by its cognate sensor HK negatively regulates its ability to induce capsular exopolysaccharide production.

**Fig 9:**
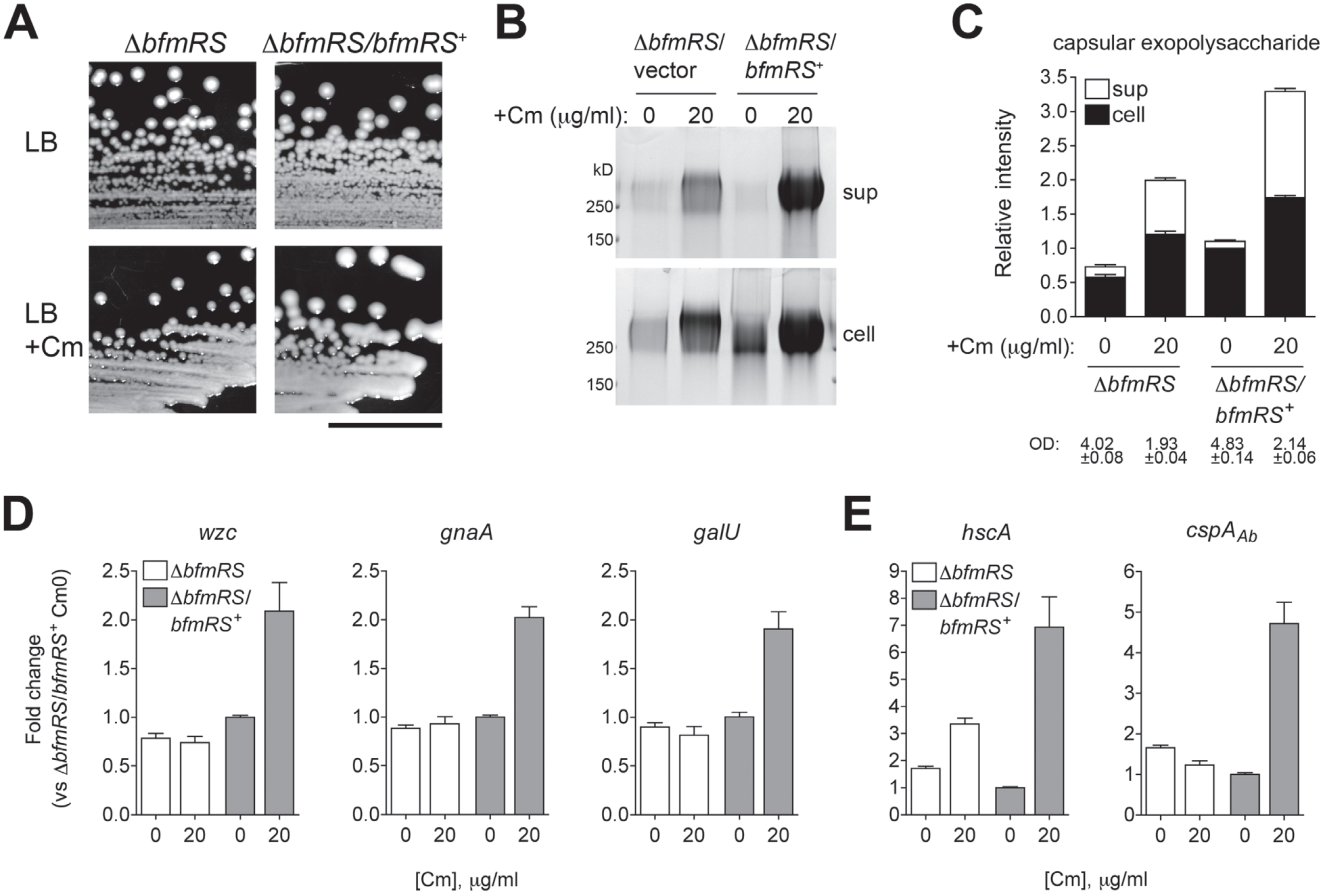
Transcriptional induction of K locus and cold shock gene expression by Cm involves BfmRS. **A**. Mucoid colony morphology on plates containing sub-MIC Cm (25 μg/ml) depends on the presence of *bfmRS*. Scale bar: 1cm. **B**. Cm at 0 or 20 μg/ml, a concentration which induces a robust transcriptional response, was added to log phase bacteria, and cell lysates and cell-free supernatants collected after overnight incubation were analyzed with alcian blue. **C**. Densitometry of stained capsule exopolysaccharide from four independent samples analyzed as in Fig. 4E. Culture OD at time of sample collection is indicated. **D, E**. Transcripts were analyzed via qRT-PCR as in Fig. 7 and fold change relative to untreated ∆*bfmRS*/*bfmRS* ^+^ was determined. Data are plotted as mean fold change ± SEM from 5 independent cultures.

We next determined whether *bfmRS* was required for exopolysaccharide induction in response to sub-MIC Cm treatment. The presence of ∆*bfmRS* blocked the hypermucoid response on plates containing Cm, while the WT *bfmRS* allele restored the response to Cm (Fig. 9A). Although there was a partial increase in capsular exopolysaccharide levels in the ∆*bfmRS* strain in the presence of Cm, the mutant lacked the robust response observed in a strain harboring an intact *bfmRS* locus (Fig. 9B, C). Transcription analysis revealed that during culture of the ∆*bfmRS* with the antibiotic there was little to no early induction of K locus gene transcripts (Fig. 9D). Induction of cold shock gene transcripts was similarly muted, although in the case of *hscA* we detected a partial response (Fig. 9E). Introduction of the WT *bfmRS* allele restored these transcriptional responses (Fig. 9D, E). These results indicate that *bfmRS* contributes to the transcriptional induction of K locus and cold shock gene expression upon exposure to sub-MIC Cm.

## Discussion

With this report, we have delineated the roles of K locus genes in the production of surface polysaccharide structures and analyzed their contributions to the growth of *A*. *baumannii* in the presence of antibiotics. We demonstrate that the high-order capsular exopolysaccharide and LPS glycoform dependent on the K locus facilitate intrinsic resistance against diverse antibiotics, consistent with their roles in providing a barrier function. Unexpectedly, our study uncovered that production of the *A*. *baumannii* capsular exopolysaccharide actively responds to antibiotic treatment. At sub-MIC, the translation inhibitors Cm and Em augment the production of capsular exopolysaccharide in a rapid and reversible manner, and strikingly this results in enhanced resistance to complement-dependent serum killing and increased virulence during disseminated infection in mice. We determined that capsular exopolysaccharide hyperproduction upon Cm exposure is a regulated process involving transcriptional increases in the expression of genes responsible for its biosynthesis and export. These changes in capsule production occur alongside regulatory changes that promote resistance to the inducing antibiotic independent of the capsule. In further support of the idea that the capsular exopolysaccharide is stress responsive, we observed that its production is also regulated by a TCS, *bfmRS*, signals from which facilitate early transcriptional induction of K locus genes by antibiotics. Antibiotic-polysaccharide interactions have multiple implications for the opportunistic nature of the pathogen, as discussed below.

These results are consistent with previous literature supporting roles for sub-MIC antibiotics in altering bacterial environmental responses [60], including defense against stressors. An earlier report with the environmental bacterium *Acinetobacter venetianus* RAG-1 showed that Cm induces the synthesis and secretion of the exopolysaccharide emulsan [61]. In that organism, the exopolysaccharide is determined by a gene cluster with similar overall organization to those in *A*. *baumannii* [62], suggesting a common mode of gene regulation upon antibiotic stress that may be a defining feature of the genus. In *E*. *coli*, a range of translation-inhibitor antibiotics including Cm, Em, and aminoglycosides augments poly-N-acetyl-glucosamine (PNAG) production and biofilm mass by a mechanism involving the second messengers ppGpp and c-di-GMP [63]. Aminoglycoside antibiotics also induce biofilm formation in some clinical strains of *P*. *aeruginosa* [64,65], although an opposite response has been observed with the macrolide azithromycin at sub-MIC [66]. In the case of *Klebsiella pneumoniae*, ciprofloxacin (a fluoroquinolone) and ceftazidime (a ß-lactam) were shown to increase capsule production [67]. It appears that across bacterial species, a range of physiological responses exists with different antibiotics that may influence behaviors important in the development and control of drug-resistant infections.

We have begun to uncover a regulatory circuit governing capsular exopolysaccharide production that involves multiple components. The BfmRS TCS appears to set the level of expression of K locus genes by controlling their transcription. According to our model, under basal conditions the transcription-promoting effects of BfmR are negatively regulated by phosphorylation signals from BfmS. Upon loss of these signals, as is the case with the *bfmS* null mutants and possibly with certain antibiotic stresses, phosphoregulation of BfmR is relieved and K locus gene expression is transcriptionally activated. Transcriptional control may account for only part of K locus gene induction upon antibiotic exposure, because a ∆*bfmRS* strain still displays a partial increase in capsular exopolysaccharide in response to sub-MIC Cm.

An additional component implicated in capsule regulation and antibiotic sensing is the ribosome. Cm and Em both target the 50S ribosomal subunit, and in doing so produce lesions in the ribosome functionally similar to that experienced with temperature downshift; it has been proposed that ribosome sensing of such a stress results in altered second messenger signaling (e.g., ppGpp) and thereby altered transcriptional responses which in *E*. *coli* mirror the cold shock response [53]. We therefore tested whether cold shock genes are similarly induced under capsule-stimulatory conditions in *A*. *baumannii*. We found that sub-MIC Cm indeed induces at least a subset of cold-shock gene homologs, and that the *bfmRS* mutant is altered in cold-shock gene expression and unable to fully induce cold shock gene transcripts early in response to sub-MIC Cm. These findings are consistent with a regulatory network controlling capsular exopolysaccharide that involves interactions between *bfmRS* and cold shock genes.

That the production of a major virulence determinant, the K locus capsular exopolysaccharide, is responsive to antibiotic stress has a possible clinical correlate in the observation that a major risk factor for opportunistic *A*. *baumannii* infections is prior or inappropriate treatment with antibiotics. We found that conditional hyperproduction of capsular exopolysaccharide induced upon exposure to sub-MIC levels of Cm allows the bacterium to overcome a key humoral defense of the host—killing by the complement system. These data add to previous work with other microorganisms on the ability of antibiotics to antagonize the bactericidal effects of serum [68]. Capsular exopolysaccharide in *A*. *baumannii* may block the function of serum complement by one or more possible means that remain to be investigated, including decreased binding of early complement components or decreased access of late complement complexes to sites required for efficient lysis. In support of the hypothesis that increased serum survival results in a more virulent organism, antibiotic-exposed bacteria hyperproducing capsular exopolysaccharide were more virulent and resulted in higher levels of bacteremia than control inocula not treated with antibiotics after intraperitoneal injection. These data are consistent with previous studies with other pathogens showing that overproduction of capsular exopolysaccharide increases virulence [69–71]. Our results are also consistent with a recent study by Bruhn and colleagues [49], which found that lethal *A*. *baumannii* disease was associated with isolates that have the ability to establish high bacterial loads in blood very early after inoculation. Our experiments involving pretreatment of bacteria with an antibiotic prior to inoculation allow an isolate that is deficient at establishing early high bacterial loads in the bloodstream to mimic the behavior of highly virulent isolates. In total, our data suggest a model wherein stimulation of enhanced capsule production, as in a patient receiving inappropriate antibiotic treatment, facilitates a transition from a low-virulence, colonizing state to one of higher virulence and invasiveness, increasing the likelihood of opportunistic disease. Whether increased protection from phagocyte killing, a prominent feature of the aforementioned studies, also contributes to the increased fitness of hyper-encapsulated *A*. *baumannii* during systemic infection is yet to be determined.

An additional consequence of hyperproduction of exopolysaccharides due to sub-MIC antibiotic exposure is that it is predicted to increase resistance of the organism against desiccation [72] and detergents [73,74]. As a consequence, exposure to low-level antibiotics should promote persistence of *A*. *baumannii* in the hospital environment. Although Cm is rarely used in developed countries, macrolides related to Em are widely used in varied settings, and are often included in empiric treatment of hospital-acquired infections for patients with multiple comorbidities or immunocompromised states [75]. Furthermore, other antibiotics more commonly administered in ICU settings such as carbapenems induce a mucoid state [76]. Therefore, inappropriate antibiotic administration may facilitate colonization in environmental reservoirs in which the organism poses the greatest risks.

In summary, these studies demonstrate a connection between regulation of the pathogenicity-determining capsular exopolysaccharide of *A*. *baumannii* and antibiotic exposure, and identify a two-component regulator that is critical for modulating this interface. These data are consistent with a model wherein environmental cues such as antibiotic stress directly alter the virulence potential of the bacterium, with possible influences on the development of nosocomial disease. Disrupting the regulatory loops that connect stress sensing with pathogenicity in *A*. *baumannii* may represent a strategy to control infections with these opportunists.

## Materials and Methods

### Bacterial strains, growth conditions, and antibiotics

Bacterial strains used in this study are listed in S1 Table. *A*. *baumannii* reference strain ATCC 17978 was used throughout unless otherwise noted. Bacteria were grown in Lysogeny Broth (LB) or on LB agar plates. Carbenicillin (100 μg/ml), gentamicin (Gm, 15 μg/ml), and/or kanamycin (Km, 30 μg/ml) were used in selection of recombinant strains. Antibiotics were purchased from Sigma. Assay subcultures of *A*. *baumannii* were grown without antibiotics in the case of chromosomal markers, and with the appropriate antibiotics in the case of plasmids. Cultures were grown at 37°C in flasks with orbital shaking at 250 RPM or in tubes rotated at maximum speed with a roller drum. Growth as optical density (OD) was monitored by measuring absorbance at 600nm.

### Molecular cloning and mutant construction

Plasmids used in this study are listed in S1 Table, and oligonucleotides (purchased from Integrated DNA Technologies) are listed in S2 Table. All constructs were sequenced (Genewiz) before introduction into *A*. *baumannii* via electroporation [77] or conjugation. The ∆*wzc*, ∆KL3, ∆*bfmS*, ∆*bfmR* and ∆*bfmRS* deletions were constructed as allelic replacements with the *aacC1* Gm^R^ cassette by amplifying 1–2kb of 17978 genomic flanking DNA, three-way ligation with pUC18 or its derivatives, insertion of the *Sac*I or *Kpn*I fragment of pFGM1 containing *aacC1*, and subcloning the resulting tripartite construct into pSR47S. pSR47S constructs were crossed into the *A*. *baumannii* chromosome via homologous recombination and Gm^R^ Km^R^ merodiploids were isolated. Gm^R^ Km^S^ double recombinants were isolated by sucrose counterselection. Markerless, in-frame deletions of *itrA* and *galU* were constructed as above, but without the insertion of *aacC1*. Km^S^ double recombinants were isolated from Km^R^ merodiploids as above. All deletion mutants were verified by colony PCR.

Point mutations were engineered in *wzc* by cloning the gene into pUC19, introducing mutations with primers covering native or engineered restriction sites via inverse PCR and self-ligation, or by amplifying and substituting a mutated gene fragment, followed by subcloning into pJB1801. Marker rescue of *wzc* was achieved by amplifying the gene and flanking regions, subcloning into pSR47S, and isolating double recombinants in EGA106 as above. Single-copy *itrA* and *galU* constructs for complementation tests were generated by cloning the genes into miniTn7 elements carried on pUC18T-miniTn7T-Gm. *itrA* was cloned with an upstream *tet*p promoter (amplified from pBR322), and *galU* was cloned with an upstream fragment containing the K locus core promoter region (amplified from 17978 genomic DNA). Insertion of the miniTn7 elements was performed by four-parental mating as described [78], but the helper plasmid pTNS3 [79] was used and exconjugants were selected on Vogel-Bonner base medium plates [80] containing Gm15. Insertion into the chromosomal *att*Tn7 site was verified by PCR as described [78]. A single-copy *bfmRS* construct for complementation tests was generated by amplifying and subcloning the operon into pEGE148. The resulting plasmid was crossed into EGA251 at the ∆*bfmRS*::*aacC1* locus.

### Sequencing of spontaneous mutants

Illumina whole-genome sequencing and analysis were performed as in [81]. Polymorphisms identified by genome sequencing of EGA2MV, EGA57, EGA127 and WT strains were used to guide PCR analysis of the corresponding loci in additional spontaneous mutants. Mucoviscous variants containing mutations in *wzc* were isolated after reviving lyophilized stocks obtained from ATCC. Mucoid bacteria containing mutations in *bfmRS* were isolated either during construction of ∆*wzc* or of unrelated recombinant strains. In the latter case, while attempting to isolate double recombinant strains from a *sacB* ^+^ merodiploid, we recovered a series of mutants that retained *sacB* but had adventitious mutations, subsequently mapped to *bfmRS*, that permitted growth on sucrose and resulted in constitutively mucoid morphology on LB plates.

### Microscopy

Bacterial capsules were visualized by the wet-film India Ink method of Duguid [82]. Images were acquired on a Zeiss Axiovert 200m microscope with 100x/1.3 lens.

### Preparation of polysaccharide samples

Extracts of *A*. *baumannii* polysaccharides from whole-cell lysates were prepared by the method of Hitchcock and Brown [83] with the following additional steps to decrease DNA and RNA content. Cells were pelleted, resuspended with lysis buffer [60mM Tris, pH 8; 10mM MgCl_2_; 50μM CaCl_2_; 20μl/ml DNase and RNase; and 3mg/ml lysozyme], and incubated at 37°C for 1 hour followed by vortexing and 3 liquid nitrogen/37°C freeze-thaw cycles. Additional DNase and RNase were added and the samples were incubated at 37°C for 30 min. SDS was added to 0.5%, followed by incubation at 37°C for an additional 30 min. The samples were then processed with boiling and proteinase K treatment as previously described [83]. Polysaccharides in culture supernatants were precipitated in 75% ice-cold ethanol overnight, followed by pelleting, air-drying, resuspending with SDS sample buffer at a volume normalized based on A_600_, and boiling for 5 minutes. Commensurate amounts of cell lysates and supernatant precipitants were loaded in SDS-PAGE.

### Electrophoresis and polysaccharide detection

Samples were separated on 4–20% BioRad TGX Tris-glycine gels and stained overnight with alcian blue as in [40]. Gels were imaged with a G-box QX4 with white-light converter (Syngene). Band intensity was quantified with GeneQuant (Syngene) via the manual band quantification method, using inter-lane spaces as background. For detection of phosphotyrosines, lysates were blotted onto PVDF membranes, incubated with 4G10 monoclonal antibodies (Millipore; 1:1000 dilution) followed by HRP-conjugated goat anti-mouse (Invitrogen), and developed with ECL-plus substrate (Perkin Elmer). Blots were stripped and re-probed with antiserum raised against *Bacillus subtilis* isocitrate dehydrogenase (ICDH).

### Antibiotic resistance assays

Bacteria grown to early post-exponential phase were normalized to OD 2, serially diluted in PBS, and spotted onto LB agar plates without antibiotic or containing serial dilutions of antibiotic. Plates were incubated at 37°C overnight and the resulting CFU were enumerated. Colony forming efficiency is defined as (# CFU on the antibiotic test plate x dilution factor)/(# CFU on the antibiotic-free plate x dilution factor).

### Serum bactericidal assays

Bacteria were grown to mid-log (OD 0.4–0.5), divided into two equal volumes to which 0 or 10 μg/ml of Cm was added, and grown for an additional 4.5 hours. Approximately 10^5^ bacteria diluted in PBS were then incubated with 30% baby rabbit complement serum (AbD Serotec) for one hour at 37°C. Control reactions were performed with serum inactivated by heating (56°C for 30 minutes). Reactions were stopped by placing on ice, and viable bacterial counts were determined by plating serial dilutions on LB agar followed by overnight incubation at 37°C.

### Animal experiments

8–10 week old female C57BL/6 mice obtained from Jackson Laboratories were used for intraperitoneal infections. Bacterial cultures were grown with or without induction by sub-MIC Cm as in serum bactericidal assays. Infections were initiated by intraperitoneal injection of approximately 10^8^ bacteria suspended in 100ul PBS into groups of mice (7–8 mice per group for survival studies; 5 per group for analyses of bacterial counts). To analyze bacterial counts, mice were euthanized after 12 hours of infection; spleens were removed aseptically and homogenized in 1ml PBS, and blood was collected via cardiac puncture followed by immediate four-fold dilution with ice-cold PBS/10mM EDTA. Viable counts were determined by plating serial dilutions as above.

### Transcript analysis

RNA was extracted via the RNeasy kit (Qiagen), DNase treated with the DNA-free kit (Ambion), and reverse transcribed with Superscript II Reverse Transcriptase (Invitrogen). cDNA was amplified with SYBR Green Master Mix (Applied Biosystems) via a StepOnePlus system according to the manufacturer’s instructions. Melting curves were obtained with each experiment to confirm reaction specificity, and reactions were performed with parallel samples lacking reverse transcriptase to verify the absence of signal from residual genomic DNA. Target amplification efficiency with each primer pair (S2 Table) was determined by obtaining a standard curve with a dilution series of cDNA and was found to be >97% in each case. Fold change in gene expression was calculated by using the 2^-∆∆Ct^ method [84] with 16S as endogenous control.

## Supporting Information

S1 Fig Analysis of growth and surface polysaccharides of K locus mutant isolates. **A, B**. Four independent ∆*wzc* isolates (isolates 9, 16, 17 and 27) were recovered after serial passaging of the merodiploid intermediate on LB without antibiotics or sucrose prior to applying sucrose counterselection. The ∆*wzc* isolates had modest defects in growth in LB media (**A**); rescue with WT *wzc*, however, did not restore growth to WT levels (**B**), suggesting the presence of second-site mutations in the original isolates. ∆*wzc* isolate 9 was not examined for growth after marker rescue because a second-site mutation was mapped to the *bfmS* locus. Cultures were grown at 37°C in a Tecan M200 Pro plate-reader. **C, D**. ∆*itrA*, ∆*galU*, and ∆KL3 mutants had WT growth kinetics. At least 3 independent isolates of each mutant were cultured independently, and A_600_ values were averaged to form each data point. Where not visible, error bars (± SEM) are within the confines of the symbol. Optical density was monitored during growth at 37°C in a Tecan M200 Pro plate-reader (**C**) or under standard culture conditions described in Methods (**D**). **E**. Analysis of polysaccharides in cell lysates separated by SDS-PAGE and stained with alcian blue. M, MW marker. Also shown are three independent ∆KL3 clones (isolates 1, 2, and 3), which were isolated directly on sucrose plates without prior passaging.
(TIF)

S2 Fig Analysis of exopolysaccharides upon sub-MIC Cm treatment. **A**. Increased capsule upon sub-MIC Cm exposure depends on KL3 genes. Exopolysaccharides were analyzed by alcian blue staining. **B**. Representative alcian blue-stained gels corresponding to panel F in Fig. 4.
(TIF)

S3 Fig Enhancement of capsular exopolysaccharide production by sub-MIC Cm treatment does not increase resistance to the heterologous antibiotic Col. WT *A*. *baumannii* were grown to mid-log phase, divided into equal volumes, and treated without or with sub-MIC Cm (10 μg/ml) for 4.5 hours to induce capsule hyperproduction. Bacteria were diluted to OD 0.02 by using spent culture supernatant filtered through 0.45 μm mixed cellulose ester membranes (Millipore) as diluent, and then challenged with serial two-fold dilutions of Col for 45 minutes at 37°C in 96-well plates. Cells were then serially diluted in PBS and plated onto LB agar to determine viable counts. Percent survival is defined as viable count after treatment with the test concentration of Col divided by the viable count without Col treatment. Data points represent the mean ± SEM from five experiments.
(TIFF)

S4 Fig Enhanced capsular exopolysaccharide production upon sub-MIC Cm treatment is not associated with altered cellular phosphotyrosine signals. Phosphotyrosine levels were determined in cells treated with 0, 10, or 30 μg/ml Cm during logarithmic growth and collected at the indicated time points (minutes). Blots were probed with the 4G10 antibody as in Fig. 2.
(TIF)

S5 Fig Effects of additional deletions within *bfmRS* locus. **A**. A *bfmS* deletion in strain 19606 causes a hypermucoid plate phenotype on LB agar similar to that seen with the 17978 background; WT 19606 colony morphology is shown in Fig. 2A. **B, C**. A *bfmR* deletion in the 17978 background is associated with plate (**B**) and India ink (**C**) phenotypes similar to that seen with the *bfmRS* double deletion (see Fig. 8); scale bars are as described in Fig. 8.
(TIF)

S1 Table Strains and plasmids used in this study. (PDF)

S2 Table Oligonucleotide primers used in this study. (PDF)
