# Supplementary figures and images for: Antibiotic Modulation of Capsular Exopolysaccharide and Virulence in Acinetobacter baumannii
Source: PLoS Pathog. 2015 Feb 13;11(2):e1004691. doi: 10.1371/journal.ppat.1004691 (PMC4334535; doi:10.1371/journal.ppat.1004691)

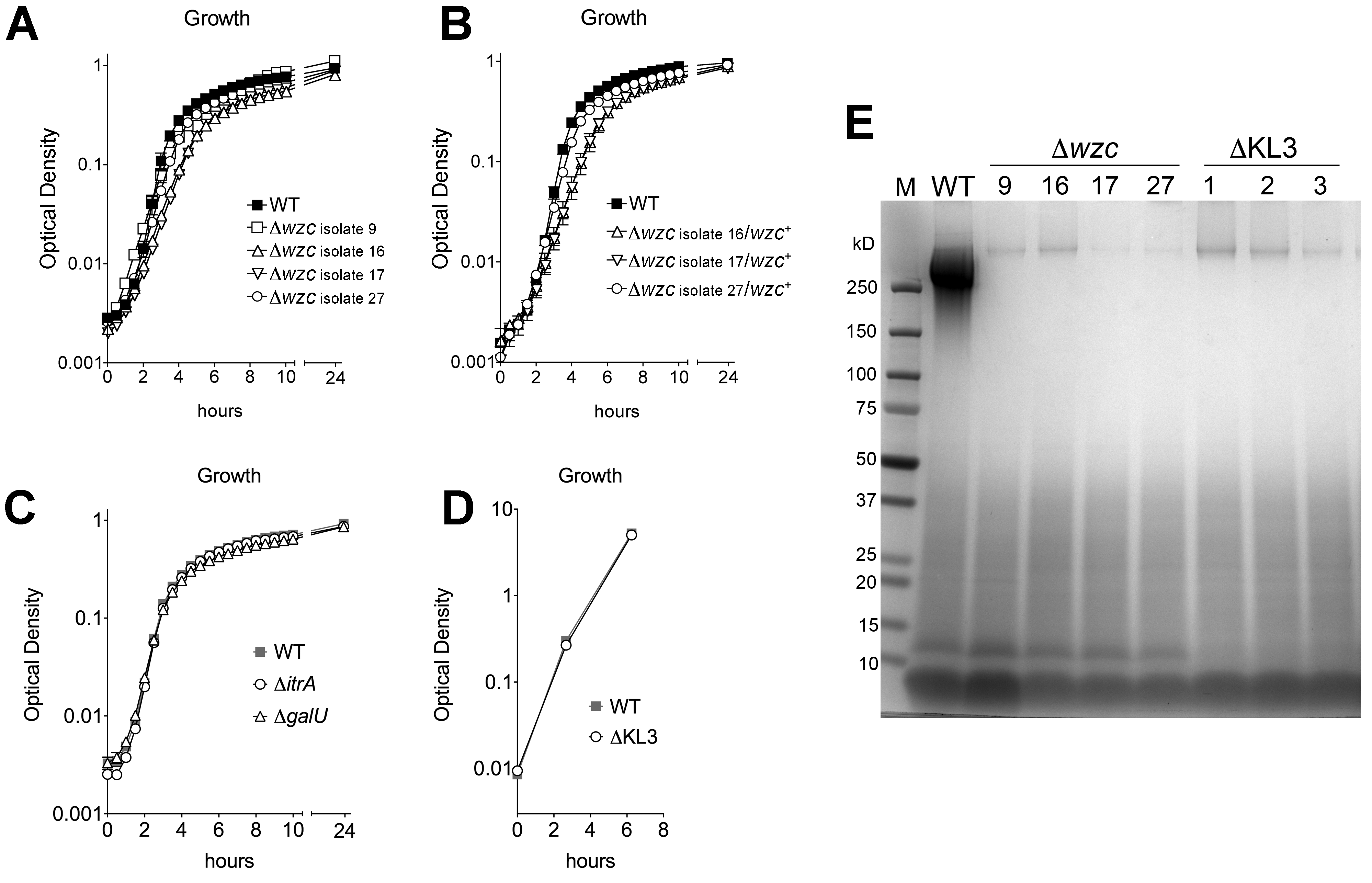

Supplement: S1 Fig — A, B. Four independent ∆wzc isolates (isolates 9, 16, 17 and 27) were recovered after serial passaging of the merodiploid intermediate on LB without antibiotics or sucrose prior to applying sucrose counterselection. The ∆wzc isolates had modest defects in growth in LB media (A); rescue with WT wzc, however, did not restore growth to WT levels (B), suggesting the presence of second-site mutations in the original isolates. ∆wzc isolate 9 was not examined for growth after marker rescue because a second-site mutation was mapped to the bfmS locus. Cultures were grown at 37°C in a Tecan M200 Pro plate-reader. C, D. ∆itrA, ∆galU, and ∆KL3 mutants had WT growth kinetics. At least 3 independent isolates of each mutant were cultured independently, and A600 values were averaged to form each data point. Where not visible, error bars (± SEM) are within the confines of the symbol. Optical density was monitored during growth at 37°C in a Tecan M200 Pro plate-reader (C) or under standard culture conditions described in Methods (D). E. Analysis of polysaccharides in cell lysates separated by SDS-PAGE and stained with alcian blue. M, MW marker. Also shown are three independent ∆KL3 clones (isolates 1, 2, and 3), which were isolated directly on sucrose plates without prior passaging. (TIF) [file ppat.1004691.s001.tif]

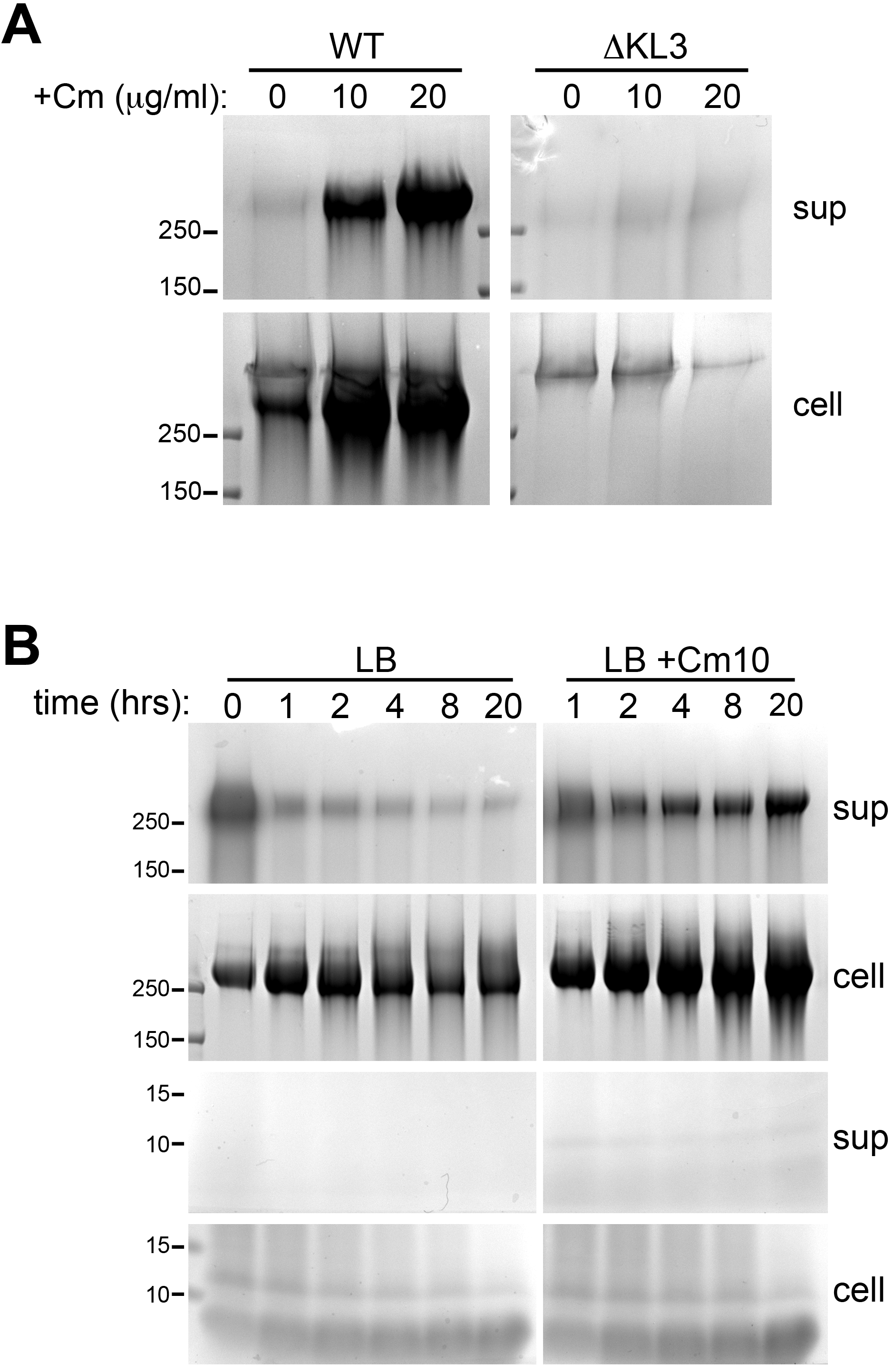

Supplement: S2 Fig — A. Increased capsule upon sub-MIC Cm exposure depends on KL3 genes. Exopolysaccharides were analyzed by alcian blue staining. B. Representative alcian blue-stained gels corresponding to panel F in Fig. 4. (TIF) [file ppat.1004691.s002.tif]

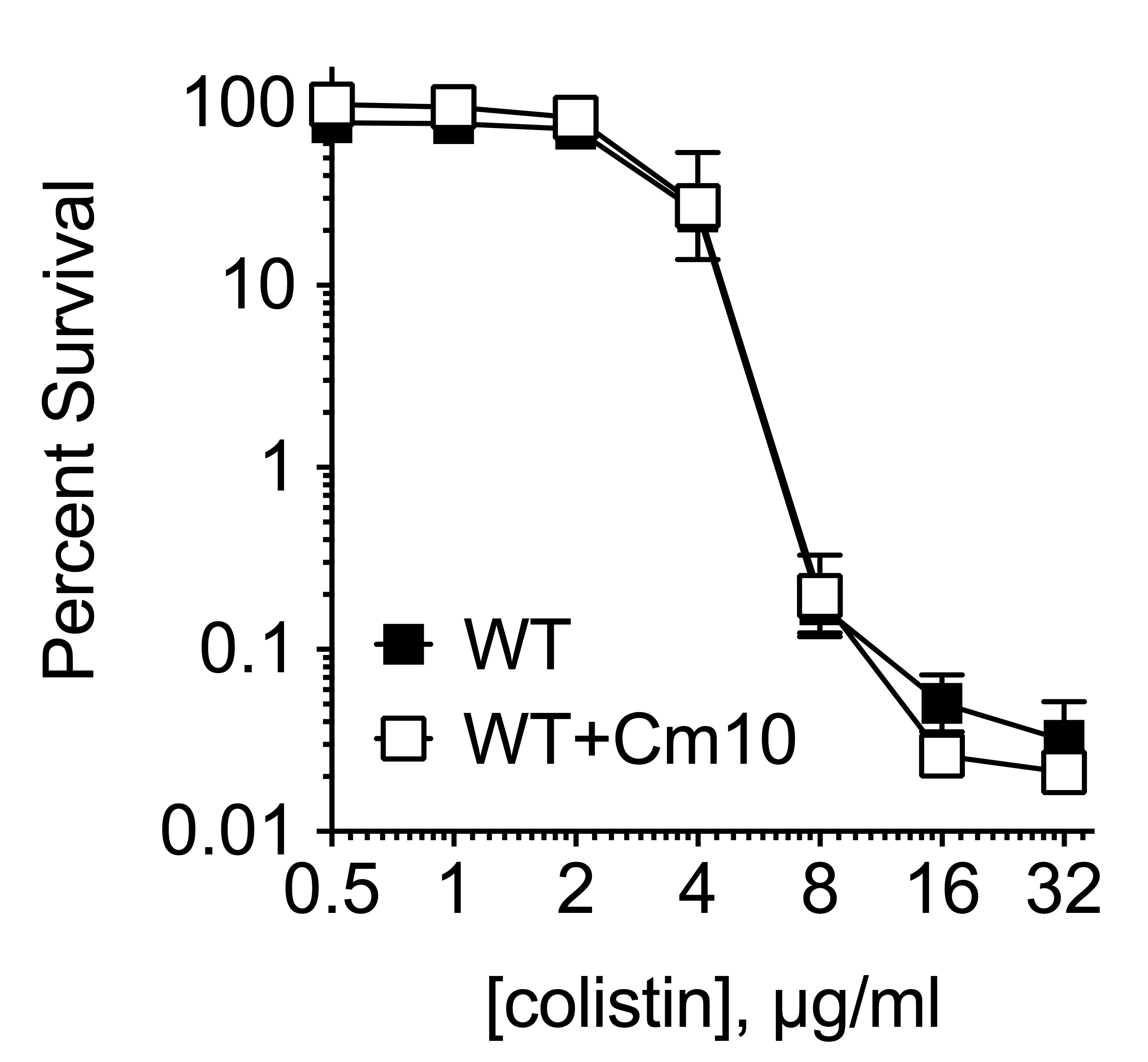

Supplement: S3 Fig — WT A. baumannii were grown to mid-log phase, divided into equal volumes, and treated without or with sub-MIC Cm (10 μg/ml) for 4.5 hours to induce capsule hyperproduction. Bacteria were diluted to OD 0.02 by using spent culture supernatant filtered through 0.45 μm mixed cellulose ester membranes (Millipore) as diluent, and then challenged with serial two-fold dilutions of Col for 45 minutes at 37°C in 96-well plates. Cells were then serially diluted in PBS and plated onto LB agar to determine viable counts. Percent survival is defined as viable count after treatment with the test concentration of Col divided by the viable count without Col treatment. Data points represent the mean ± SEM from five experiments. (TIFF) [file ppat.1004691.s003.tiff]

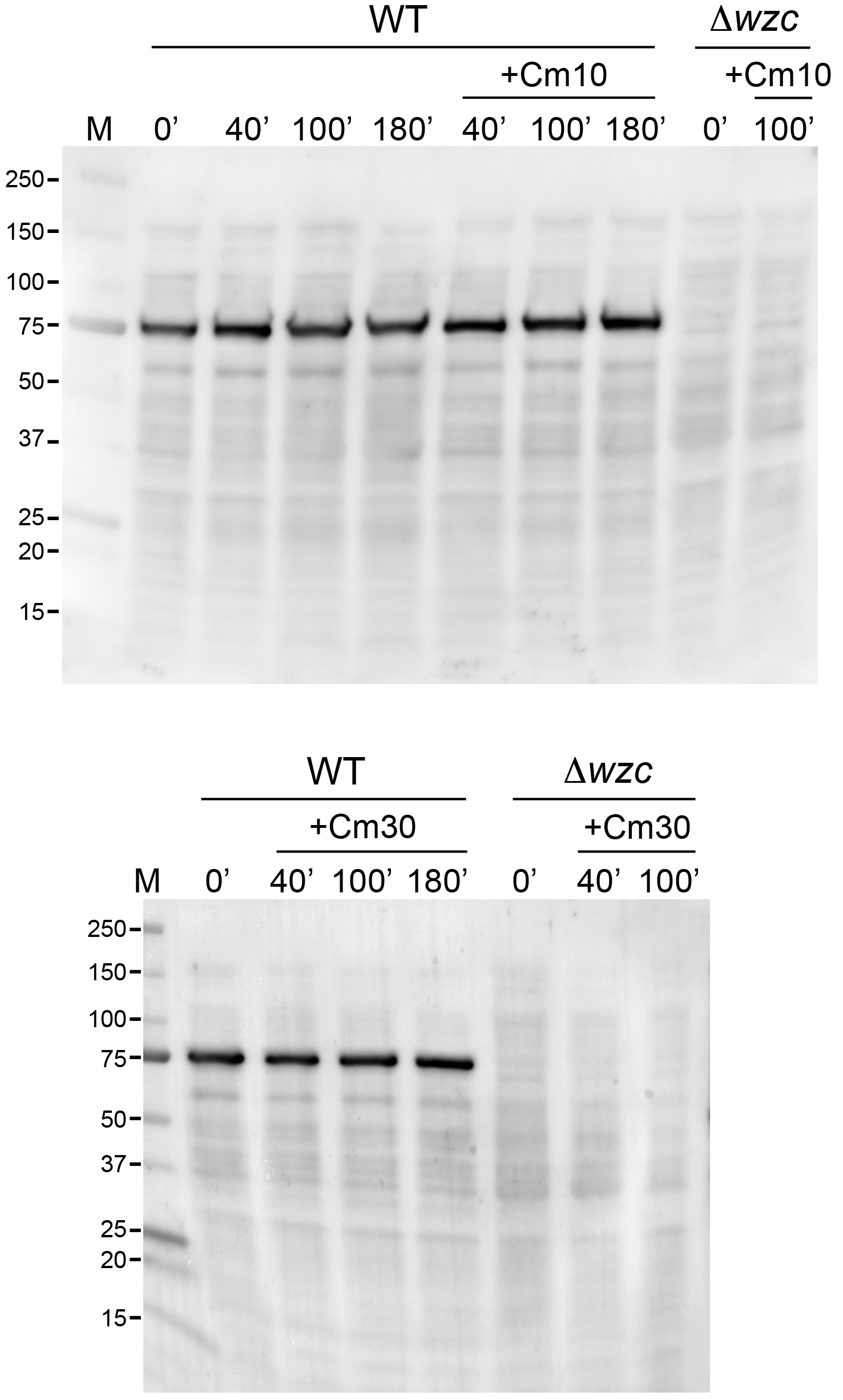

Supplement: S4 Fig — Phosphotyrosine levels were determined in cells treated with 0, 10, or 30 μg/ml Cm during logarithmic growth and collected at the indicated time points (minutes). Blots were probed with the 4G10 antibody as in Fig. 2. (TIF) [file ppat.1004691.s004.tif]

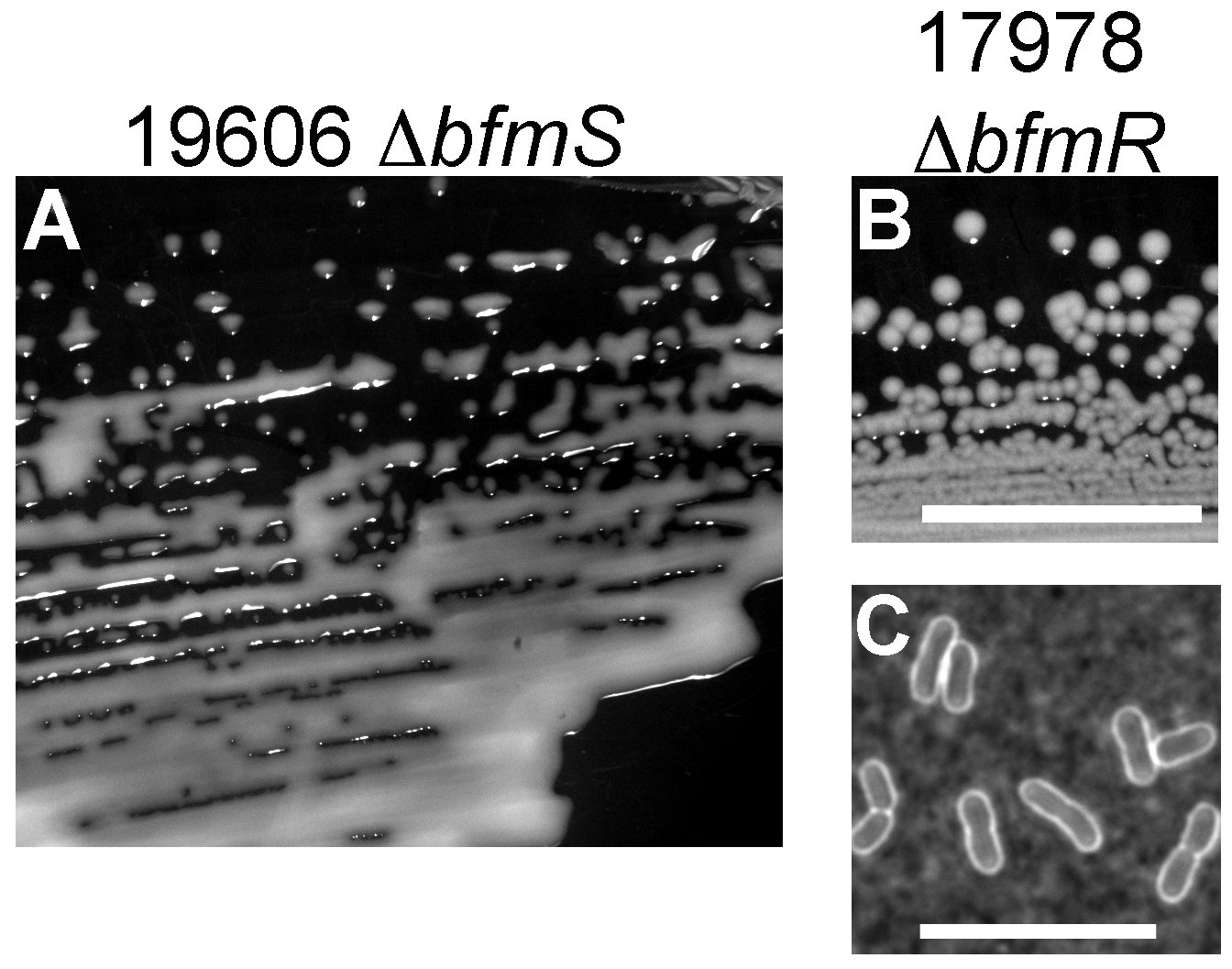

Supplement: S5 Fig — A. A bfmS deletion in strain 19606 causes a hypermucoid plate phenotype on LB agar similar to that seen with the 17978 background; WT 19606 colony morphology is shown in Fig. 2A. B, C. A bfmR deletion in the 17978 background is associated with plate (B) and India ink (C) phenotypes similar to that seen with the bfmRS double deletion (see Fig. 8); scale bars are as described in Fig. 8. (TIF) [file ppat.1004691.s005.tif]
