# Supplementary material for: Antibiotic Modulation of Capsular Exopolysaccharide and Virulence in Acinetobacter baumannii
Source: PLoS Pathog. 2015 Feb 13;11(2):e1004691. doi: 10.1371/journal.ppat.1004691 (PMC4334535; doi:10.1371/journal.ppat.1004691)
Supplement: S1 Table — (PDF) [file ppat.1004691.s006.pdf]

Table S1. Strains and plasmids used in this study.

Strain or plasmid	Genotype or description	Reference
A. baumannii		
ATCC 17978	cerebrospinal fluid isolate	[1]
ATCC 19606	urine isolate	[1]
ATCC 17961	blood isolate	[1]
EGA70	mucoviscous derivative of ATCC 17978, *ptk*(G546C)	this work
EGA2MV	mucoviscous derivative of ATCC 19606, *ptk*(D569N)	this work
EGA57	mucoviscous derivative of ATCC 17961, *ptk*(V545E)	this work
EGA106	ATCC 17978 Δ*ptk*::*aacC1*	this work
EGA263	*ptk*$^+$ marker rescue of EGA106	this work
EGA108	ATCC 17978 Δ*ptk*::*aacC1* with pJB1801	this work
EGA110	ATCC 17978 Δ*ptk*::*aacC1* with pEGE98	this work
EGA109	ATCC 17978 Δ*ptk*::*aacC1* with pEGE97	this work
EGA111	ATCC 17978 Δ*ptk*::*aacC1* with pEGE99	this work
EGA170	ATCC 17978 Δ*ptk*::*aacC1* with pEGE106	this work
EGA122	ATCC 17978 Δ*ptk*::*aacC1* with pEGE91	this work
EGA171	ATCC 17978 Δ*ptk*::*aacC1* with pEGE107	this work
EGA168	ATCC 17978 Δ*ptk*::*aacC1* with pEGE104	this work
EGA169	ATCC 17978 Δ*ptk*::*aacC1* with pEGE105	this work
EGA298	ATCC 17978 with miniTn7T-Gm	this work
EGA295	ATCC 17978 Δ*itrA* in-frame deletion	this work
EGA301	ATCC 17978 Δ*itrA* miniTn7T-Gm::*itrA*	this work
EGA294	ATCC 17978 Δ*galU* in-frame deletion	this work
EGA308	ATCC 17978 Δ*galU* miniTn7T-Gm::*galU*	this work
EGA68	ATCC 17978 ΔKL3::*aacC1*	this work
EGA195	ATCC 17978 Δ*bfmS*::*aacC1*	this work
EGA250	ATCC 17978 Δ*bfmR*::*aacC1*	this work
EGA251	ATCC 17978 Δ*bfmRS*::*aacC1*	this work
EGA127	ATCC 17978 *bfmS*$^{1\text{-}467}$(G467DfsX19)	this work
EGA216	ATCC 19606 Δ*bfmS*::*aacC1*	this work
EGA280	EGA251 *aacC1*::pEGE148	this work
EGA281	EGA251 *aacC1*::pEGE152	this work
E. coli		
DH5α	*supE44* Δ*lacU169* (φ80lacZΔM15) *hsdR17 recA1 endA1 gyrA96 thi*-1 *relA1*	[2]
DH5λpir	DH5α (λpir) *tet*::Mu *recA*	[3]
TO60	DH5α (λpir) [F′ *proAB lacI*^{q}ZΔM15 Tn10 (tet^R)]	[4]
**plasmids**		
pUC18	*oriColE1* MCS Amp^R	[5]
pJB1801	*oriRSF1010* Amp^R	Gift of J. Vogel
pEGE98	pJB1801::*ptk*	this work
pEGE97	pJB1801::*ptk*(V545E)	this work
pEGE99	pJB1801::*ptk*(G546C)	this work
pEGE106	pJB1801::*ptk*(K547Q)	this work
pEGE91	pJB1801::*ptk*(D569N)	this work
pEGE107	pJB1801::*ptk*(D649N)	this work

pEGE104	pJB1801::*ptk*($Y_{1,2,3,4,5,6}$F)	this work
pEGE105	pJB1801::*ptk*($Y_{1,3,5}$F)	this work
pFGM1	Amp^R Gm^R; source of *aacC1* cassette	[6],Gift of H. Schweizer
pSR47s	*oriTRP4 oriR6K* Km^R	[7]
pEGE76	pSR47s containing Δ*ptk*::*aacC1* deletion construct	this work
pEGE138	pSR47s::ptk^+ (marker rescue allele)	this work
pEGE80	pSR47s containing ΔKL3 deletion allele	this work
pEGE181	pSR47s containing Δ*itrA* in-frame deletion construct	this work
pEGE179	pSR47s containing Δ*galU* in-frame deletion construct	this work
pEGE125	pSR47s containing Δ*bfmS*::*aacC1* deletion construct	this work
pEGE132	pSR47s containing Δ*bfmR*::*aacC1* deletion construct	this work
pEGE133	pSR47s containing Δ*bfmRS*::*aacC1* deletion construct	this work
pEGE148	pSR47s::*aacC1* Km^R Gm^R	this work
pEGE152	pEGE148::*bfmRS*	this work
pUC18T-miniTn7T-Gm	*oriColE1* Amp^R; miniTn7 base vector	[6], Gift of H. Schweizer
pEGE193	pUC18T-miniTn7T-Gm::KL3p-*galU*	this work
pEGE194	pUC18T-miniTn7T-Gm::*tet*p-*itrA*	this work
pTNS3	helper plasmid carrying Tn7 transposase	[8], Gift of H. Schweizer

References

1. Bouvet P, Grimont P (1986) Taxonomy of the Genus Acinetobacter with the Recognition of Acinetobacter baumannii sp. nov. Acinetobacter haemolyticus sp. nov. Acinetobacter johnsonii sp. nov. and Acinetobacter junii sp. nov. and Emended Descriptions of Acinetobacter calcoaceticus and Acinetobacter lwofii. International Journal of Systematic Bacteriology 36: 228-240.
2. Hanahan D, Jessee J, Bloom FR (1991) Plasmid transformation of Escherichia coli and other bacteria. Methods Enzymol 204: 63-113.
3. Kolter R, Inuzuka M, Helinski DR (1978) Trans-complementation-dependent replication of a low molecular weight origin fragment from plasmid R6K. Cell 15: 1199-1208.
4. O'Connor TJ, Adepoju Y, Boyd D, Isberg RR (2011) Minimization of the Legionella pneumophila genome reveals chromosomal regions involved in host range expansion. Proc Natl Acad Sci U S A 108: 14733-14740.
5. Yanisch-Perron C, Vieira J, Messing J (1985) Improved M13 phage cloning vectors and host strains: nucleotide sequences of the M13mp18 and pUC19 vectors. Gene 33: 103-119.
6. Choi KH, Gaynor JB, White KG, Lopez C, Bosio CM, et al. (2005) A Tn7-based broad-range bacterial cloning and expression system. Nat Methods 2: 443-448.
7. Andrews HL, Vogel JP, Isberg RR (1998) Identification of linked Legionella pneumophila genes essential for intracellular growth and evasion of the endocytic pathway. Infect Immun 66: 950-958.
8. Choi KH, Mima T, Casart Y, Rholl D, Kumar A, et al. (2008) Genetic tools for select-agent-compliant manipulation of Burkholderia pseudomallei. Appl Environ Microbiol 74: 1064-1075.
