# Supplementary material for: Antibiotic Modulation of Capsular Exopolysaccharide and Virulence in Acinetobacter baumannii
Source: PLoS Pathog. 2015 Feb 13;11(2):e1004691. doi: 10.1371/journal.ppat.1004691 (PMC4334535; doi:10.1371/journal.ppat.1004691)
Supplement: S2 Table — (PDF) [file ppat.1004691.s007.pdf]

Table S2. Oligonucleotide primers used in this study.

primer name	Sequence (5' – 3'; restriction site underlined)	RE site
cloning		
Dptk17-dn-F	TTTAGGTACCTTGTATCTTCGGTATTAGTATTTTGTTGG	KpnI
Dptk-dn-R	TTAAGTCGACTGCTTTTTGAAGCTCTTCTGG	SalI
Dptk-up-R	CTATGGTACCGCCTATAAGGCACAAAAAGAAGAC	KpnI
Dptk-up-F	TGGTGCGGCCGCTTTACGCGATACACAAATGACAG	NotI
DitrA-upF	CTAAGTCGACTCCCTTTTCTATCGCTTCG	SalI
DitrA-upR	CTATGGTACCTAAACGCTTCATATTTTCATTCACTC	KpnI
DitrA-dwnF	AGAAGGTACCGATTCTTCTGTTAAGTAAATTTTATATCTTG	KpnI
DitrA-dwnR	TAGTGGATCCAGCATAACCGATTCAACACC	BamHI
DgalU-upR	TACCGGTACCGGCTACTGGTAAAACTGC	KpnI
DgalU-upF	TGTTTGTCGACAAGATGGCCCTTTAACTCCTG	SalI
DgalU-dwnF	ACTTGGTACCTAGAATTTTGTAGCTCTGCTTAACTATTTG	KpnI
DgalU-dwnR	TACCGGATCCTTTTGAAAGCGGTCTTGCTC	BamHI
DKL3-dn-F	ATGTGGTACCATCTTTATTTAAAGGTAATGATACCCAAG	KpnI
DKL3-dn-R	TCATGTCGACAGTTTTCACCACCAAATCCAACAC	SalI
galU-F	AATGGGATCCAAGAAAATATTGATTCTTCTGTTAAG	BamHI
galU-R	TCGTGGTACCAAATAGTTAAGCAGAGCTACAAAATTC	KpnI
itrA-F	AACAGGATCCTTCTGGTACGGTCTGCGTTTAG	BamHI
itrA-R	TACCGGTACCGGCTACTGGTAAAACTGC	KpnI
KL3-IG-R	GCAAGGGATCCAAAAGTTAGCCTTTTTCCTTACAAGATT	BamHI
KL3-IG-F	AATAGAGCTCACGCGACCCTTTGTGTATTTATC	SacI
ptet F	AAATGAGCTCATCACGAGGCCCTTTCGTCTTC	SacI
ptet R	GATTGGATCCACGGTGCCTGACTGCGTTAGC	BamHI
ptk17-F	CCATTGGATCCATAAAAATGTGCCCGATCCTTAC	BamHI
ptk-R	AAGCGTCGACGTCTGCTTTACGTCCGACTTCTG	SalI
YF1-6ptk-F	ACGTGCTAGCGCTGGCTTTGGTTTTGGCTTTAACTTTGCTTTTGCCTTT<br>AAGGCACAAAAAGAAGACTAATTAAAACTC	NheI
YF135ptk-F	ACGTGCTAGCGCTGGCTTTGGTTATGGCTTTAACTATGCTTTTGCCTAT<br>AAGGCACAAAAAGAAGACTAATTAAAACTC	NheI
K547Q-F	CTTCTCCGGAAGTGGGTCAATCGTTTATTTCAACTAACTTGG	BspEI
K547Q-R	ACCCACTTCCGGAGAAGGACCAGCAATC	BspEI
D649N-F	AATACTCCACCAGTGCTTGCAGTAAC	
D649N-R	GATAATGATGTGGTCATATTGGCTTTG	
DbfmS-upF	AGCAGGATCCGTGAAAGCAAATCAAGCTCTACACC	Bam
DbfmS-upR	TACTGAGCTCAAACACTCGACCAACCTTATAGGAAG	SacI
DbfmS-dnF	AGCAGAGCTCTTTAAACAACCGCCATTAAAGACC	SacI
DbfmS-dnR	CTATGTCGACTTATTGGAATTGCATCTCGTCCTC	SalI
DbfmR-upF	TCAAGGATCCGTTGCATAAATAGGTCAGGAACC	Bam
DbfmR-upR	CTTTGAGCTCTTTTCTTCTTGGCTCATATCATTGC	SacI
DbfmR-dnF	CAATGAGCTCTAAAATCTGATTAAACTTCCTATAAGGTTGG	SacI
DbfmR-dnR	AAAGCGCGGCCGCTAAACACCCAGATGCAGATAATGC	NotI
bfmR-F	TCTGGGATCCTTGTCGGGAGATAGCATACCAAAG	Bam
bfmS-R	CTGAAAGCTTGCAAACCTATTTTGGAACCTGATG	HindIII
qRT-PCR		
ptk-qF	CTCCACCAGTGCTTGCAGTA	
ptk-qR	CAGCGCTAGCACGTTGAATA	

52_qF2	GCAGCGGGTAGTAAGTGGAA	
52_qR2	GCAACATAGCGCGCCATATT	
galU-qF	AGCCAAGCTGCTCAAATCAT	
galU-qR	CGGCCAACCACAGATAAGTT	
hscA-qF	AGGCAGATACGGAACGCTTA	
hscA-qR	ATGATCCCTTGGCAGAGTTG	
csp0767-qF	ACCGGATGTTCAGCTTTCTG	
csp0767-qR	ACAAACCAACGTGCCTCTTC	
16S_qF	CAGCTCGTGTCGTGAGATGT	
16S_qR	CGTAAGGGCCATGATGACTT	
